# Understanding the [NiFe] Hydrogenase Active Site Environment through Ultrafast Infrared and 2D-IR Spectroscopy of the Subsite Analogue K[CpFe(CO)(CN)_2_] in Polar and Protic Solvents

**DOI:** 10.1021/acs.jpcb.3c07965

**Published:** 2024-02-01

**Authors:** Barbara Procacci, Solomon L. D. Wrathall, Amy L. Farmer, Daniel J. Shaw, Gregory M. Greetham, Anthony W. Parker, Yvonne Rippers, Marius Horch, Jason M. Lynam, Neil T. Hunt

**Affiliations:** † Department of Chemistry, York Biomedical Research Institute, University of York, York YO10 5DD, U.K.; ‡ STFC Central Laser Facility, Research Complex at Harwell, Rutherford Appleton Laboratory, Harwell Campus, Didcot OX11 0QX, U.K.; § Department of Physics, Ultrafast Dynamics in Catalysis, Freie Universität Berlin, Arnimallee 14, 14195 Berlin, Germany

## Abstract

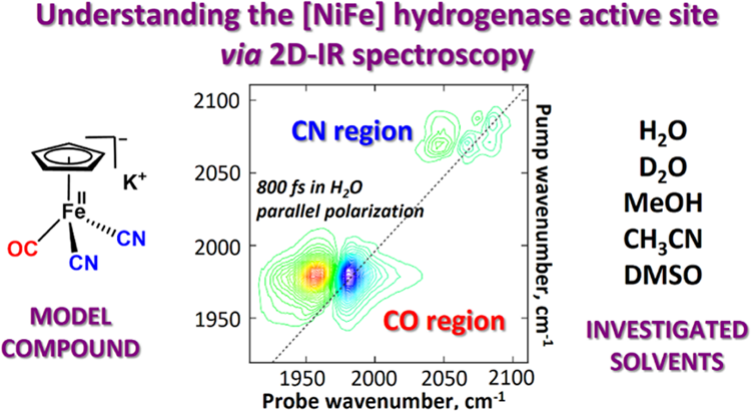

The [CpFe(CO)(CN)_2_]^−^ unit is an excellent structural model for the Fe(CO)(CN)_2_ moiety of the active site found in [NiFe] hydrogenases. Ultrafast infrared (IR) pump–probe and 2D-IR spectroscopy have been used to study K[CpFe(CO)(CN)_2_] (**M1**) in a range of protic and polar solvents and as a dry film. Measurements of anharmonicity, intermode vibrational coupling strength, vibrational relaxation time, and solvation dynamics of the CO and CN stretching modes of **M1** in H_2_O, D_2_O, methanol, dimethyl sulfoxide, and acetonitrile reveal that H-bonding to the CN ligands plays an important role in defining the spectroscopic characteristics and relaxation dynamics of the Fe(CO)(CN)_2_ unit. Comparisons of the spectroscopic and dynamic data obtained for **M1** in solution and in a dry film with those obtained for the enzyme led to the conclusion that the protein backbone forms an important part of the bimetallic active site environment via secondary coordination sphere interactions.

## Introduction

**Figure 1:**
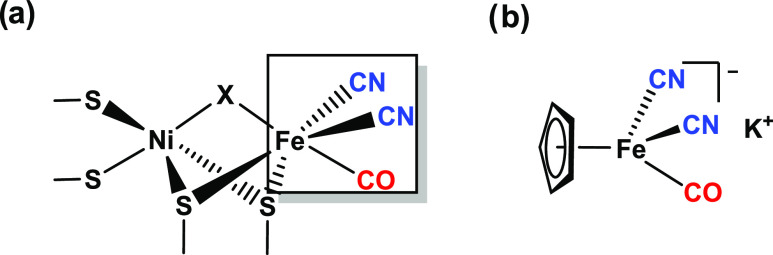
(a) Diagram of the generalized active site structure of [NiFe] hydrogenases. (b) Structure of the structural model compound **M1** employed in this study, oriented for ease of comparison.

[NiFe] hydrogenases catalyze the interconversion of protons with dihydrogen, and so the mechanistic processes underpinning this transformation have become the focus of considerable interest, with a view to informing future biomimetic or biotechnological approaches to sustainable fuel generation. The active site of [NiFe] hydrogenases features a bimetallic structure (Figure 1a) in which Ni and Fe atoms are bridged by the sulfur atoms of two cysteine residues, while the Ni is covalently linked to the protein scaffold via two further terminally coordinated cysteines.^1−4^ The Fe center is coordinated by two terminal CN ligands and one terminal CO ligand. The results of crystallographic studies indicate that hydrogen bonds exist between the two CN ligands and side chains of nearby protein residues.^5−9^ Although the catalytic mechanism of the [NiFe] hydrogenases involves a number of redox and structural changes of the [NiFe] center, the Fe(CO)(CN)_2_ unit remains intact, and the Fe atom retains the low spin, Fe(II) oxidation state throughout.^1^

While the structure of the active site and the various redox-structural states involved in the reaction mechanism have been the topic of much research and so are better understood,^1,2,7,10−12^ the dynamic nature of the active site, relating both to the transitions between states and to the structural dynamics of the enzyme at equilibrium, is less well documented.^13−19^ Recently, ultrafast infrared (TRIR) and 2D-IR spectroscopy have been applied to study the CO and CN stretching modes (ν_CO_, ν_CN_) of the [NiFe] center from three separate organisms.^20−22^ These studies all reached similar conclusions: the enzyme seems to create a remarkably rigid environment around the Fe(CO)(CN)_2_ unit. This is based on the observation that the vibrational spectra show little inhomogeneous line broadening and there is no evidence of the rapid structural dynamics normally associated with organometallic compounds in solution.^23−34^ Furthermore, studies of the vibrational relaxation times of the first excited vibrational states (*v* = 1) of the ν_CO_ and ν_CN_ modes show that the active site is free of bulk water, as also suggested by crystallography experiments.^6^

One of the key advantages of ultrafast infrared spectroscopy methods is the ability to probe the precise nature of the vibrational potential energy surfaces of the ν_CO_ and ν_CN_ modes by accessing higher-lying vibrational levels. When compared to absorption spectroscopy, this provides detailed information about the nature of the bonding of the ligands and the vibrational coupling and energy-transfer processes occurring in the active site. Such information is vital in benchmarking quantum mechanical models of the active site and will lead to an enhanced understanding of the nature of the biological molecule and improve our ability to predict the details of transitions between intermediates along the reaction coordinate. In the case of the regulatory hydrogenase from *Cupriavidus necator* formerly known as *Ralstonia eutropha*; (*Cn*RH) and hydrogenase-1 from *Escherichia coli* (*Ec*Hyd1), 2D-IR spectroscopy demonstrated that the ν_CO_ and ν_CN_ modes were weakly mutually coupled,^20,22^ consistent with data from absorption spectroscopy on isotopically modified enzymes,^35−37^ and the two ν_CN_ modes were extremely strongly coupled. In addition, the two ν_CN_ modes exhibited a marked difference in anharmonicity, with the higher-frequency symmetric stretching mode possessing a much smaller value (8 cm^–1^) than the lower-frequency antisymmetric stretching mode (18–20 cm^–1^). The origin of this disparity remains to be conclusively identified experimentally. Of note is that similar effects have not been observed in organometallic systems with CO or CN ligands in the solution phase, yet it seems to be a consistent feature of the small number of enzyme active sites for which the ν_CN_ potential energy surfaces have already been investigated.^20^ This raises the possibility that the enzyme scaffold has a role to play in defining the nature of the active site. However, an alternative explanation, from recent studies using the generalized second-order vibrational perturbation theory on a density functional theory (DFT) level, has suggested that the feature could also be a first coordination sphere effect intrinsic to the Fe(CO)(CN)_2_ unit arising from a 2–2 Darling–Dennison resonance.^38^

To shed further light on the nature of the spectroscopy and potential energy surfaces of the Fe(CO)(CN)_2_ unit and to establish the precise impact of the local environment upon both its spectroscopy and equilibrium dynamics, we now report a series of ultrafast IR pump–probe and 2D-IR spectroscopy experiments on model compound K[CpFe(CO)(CN)_2_] (**M1**) (Figure 1b) in a range of solvents and as a dry film. The preparation and structure of **M1** have been reported previously alongside a detailed analysis of the fundamental IR vibrational frequencies of the ν_CO_ and ν_CN_ modes.^37,39^ It was concluded that not only was the CpFe(CO)(CN)_2_ moiety an excellent structural mimic of the Ni(μ-SCys)_2_Fe(CO)(CN)_2_ unit of the enzyme active site but also that it provides an almost exact infrared spectral analogue of as-isolated hydrogenases from *Chromatium vinosum* and *Desulfovibrio gigas*.^37,39^ This similarity motivated a detailed study of the solvent dependence of the vibrational frequencies of **M1** with a particular focus on the role of hydrogen bonding in defining the ν_CO_ and ν_CN_ stretching mode frequencies. Here, we seek to build on this work, which established a basis for interpreting IR absorption spectra of the [NiFe] hydrogenases by using **M1** to provide a similar benchmark for ultrafast spectroscopy studies of the enzyme active sites. Measurements of anharmonicity, intermode coupling strength, vibrational relaxation time, and solvent dynamics of the ν_CO_ and ν_CN_ modes of **M1** in H_2_O, D_2_O, methanol, dimethyl sulfoxide, and acetonitrile reveal that the local environment, including H-bonding to the CN ligands, plays an important role in defining the spectroscopic characteristics accessible via ultrafast methods, including mode anharmonicities and relaxation behavior of the ν_CO_ and ν_CN_ stretching modes of the Fe(CO)(CN)_2_ moiety. These studies also reinforce the observation that the molecular dynamics observed in solution environments represent a poor model for those in the enzyme and provide insight into the manner in which the protein creates a very specific local environment for the catalytic center.

## Methods

### Synthesis and Characterization of K[CpFe(CO)(CN)_2_]

The complex **M1** was synthesized and isolated using a modified published procedure.^40^ An additional purification step was performed at the end of the reported synthesis by dissolving the complex in CH_3_CN and leaving it to stir for 10 min. The resulting solution was then filtered and dried under vacuum. IR (ν_CO_ 1978 cm^–1^, ν_CN1_ 2067 cm^–1^, ν_CN2_ 2084 cm^–1^ in H_2_O); mass spec (ESI neg, *m*/*z*): 201 (M^–^) exp 200.9756 calc’d for C_8_H_5_FeN_2_O 200.9757 difference 0.1 mDa; NMR (CD_3_OD): ^1^H 4.70 (s, 5H); ^13^C 83.2 (s, Cp), 154.0 (s, CO), 220.4 (s, CN); (D_2_O): ^1^H 4.85 (s, 5H); ^13^C 83.0 a (s, Cp), 160.5 (s, CO), 218.0 (s, CN); ^1^H-^13^C HMQC displayed cross peaks between the reported frequencies in both solvents (see the Supporting Information for spectrum in D_2_O).

**Table 1:** Data Obtained from IR Absorption, IR Pump–Probe, and 2D-IR Spectroscopy of **M1**^a^

	FT-IR			pump–probe						2D-IR			
				T_1_, ps			anisotropy, ps			intramode anharmonicity			spectral diffusion
	v(CO) (fwhm)	v(CO) (fwhm)	v(CN_2_) (fwhm)	CO	CN_1_	CN_2_	CO	CN_1_	CN_2_	CO	CN_1_	CN_2_	
H_2_O	1978 (20)	2067 (14)	2084 (12)	5 ± 1	6 ± 1	8 ± 1	4 ± 1	2 ± 1	6 ± 4	24	22	10	0.51 ± 0.03
D_2_O	1978 (20)	2067 (14)	2084 (12)	24 ± 1	34 ± 1	44 ± 2	9 ± 1	3 ± 1	7 ± 1	24	22	10	0.71 ± 0.03
MeOH	1972 (17)	2083 (12)	2094 (12)	21 ± 1	40 ± 3	57 ± 3	9 ± 1	0.6 ± 0.1	0.3 ± 0.1	25	19	16	2.1 ± 0.5
CH_3_CN	1952 (22)	2088 (8)	2094 (8)	16 ± 1	67^b^ ± 5		8 ± 1	1.4^b^ ± 0.3		25	23	18	
DMSO	1943 (22)	2086 (8)	2094 (7)	17 ± 1	147^b^ ± 14		11 ± 1	1.0^b^ ± 0.1		25	23	20	1.55 ± 0.09
dry film	1970 1950	2083 2087	2093 2097	15–18	135					17	17	2	
as-isolated EcHyd1	1908 1922 (5–12)	2057 2050 (5)	2070 2063 (5)	16–25	30–40					25	20	8	
ReRH	1943 (7)	2071 (6)	2080 (6)	18	30					25	18	8	

a IR frequencies (FWHM, cm^–1^), vibrational lifetimes (ps), anisotropy decay time scales (ps), intramode anharmonicities (cm^–1^), and spectral diffusion time scales (ps) are listed for the v_CO_ and v_CN_ modes of **M1** in the solvents studied and the dry film. Values obtained for as-isolated EcHyd1^20^ and CnRH^22^ are reported for comparison.

b The two CN modes are very close in frequencies for these solvents, yielding corresponding pump–probe bleaches that overlap within the resolution of the instrument. As a consequence, a single lifetime has been obtained for the two modes.

### Sample Preparation for IR Spectroscopy

The samples for all IR spectroscopy experiments were prepared by placing 50 μL of a 2.5 mM solution of **M1** into a transmission cell (Harrick) featuring two CaF_2_ windows separated by a PTFE spacer (50 or 100 μm) to specify the optical path length.

### IR Absorption Spectroscopy

IR absorption spectra were recorded in transmission mode at room temperature using a Bruker Vertex 70 FT-IR spectrometer with a spectral resolution of 2 cm^–1^. The empty spectrometer under a N_2_ atmosphere was used to acquire background spectra for reference. All spectra reported were the average of 20 scans.

### Ultrafast IR Spectroscopy

Ultrafast spectroscopy experiments were performed using the ULTRA laser system as reported previously.^41^ Mid-IR pulses with a central frequency of 2000 cm^–1^, a bandwidth in excess of 300 cm^–1^, a pulse duration of 50 fs, and a 10 kHz repetition rate were used in all cases.

IR pump–probe spectra were recorded by scanning the pump–probe delay time (*T*_w_) from −20 to 150 ps. Each experiment was performed by using both parallel and perpendicular pump–probe polarization geometries.

2D-IR spectra were acquired using the pseudo-pump–probe method.^41−43^ In brief, pump-pulse pairs were created using a mid-IR pulse shaper, applying a four-frame phase cycling.^43−46^ The coherence time (τ) of the pair of collinear pump pulses was scanned in increments of 30 fs from 0 to 3 ps, and spectra were recorded at *T*_w_ values of 125, 250, 500, 750, 1, 3, 15, and 45 ps. The pump frequency axis was generated by Fourier transformation of the time domain data with respect to τ. The probe frequency axis was generated by dispersing the signal with a spectrograph followed by detection with liquid nitrogen-cooled 128-element mercury–cadmium-telluride (MCT) detectors, giving a frequency resolution of <2 cm^–1^. Spectra at each *T*_w_ were acquired by using both parallel and perpendicular pump–probe polarization relationships.

## Results

### IR Absorption Spectroscopy

**Figure 2:**
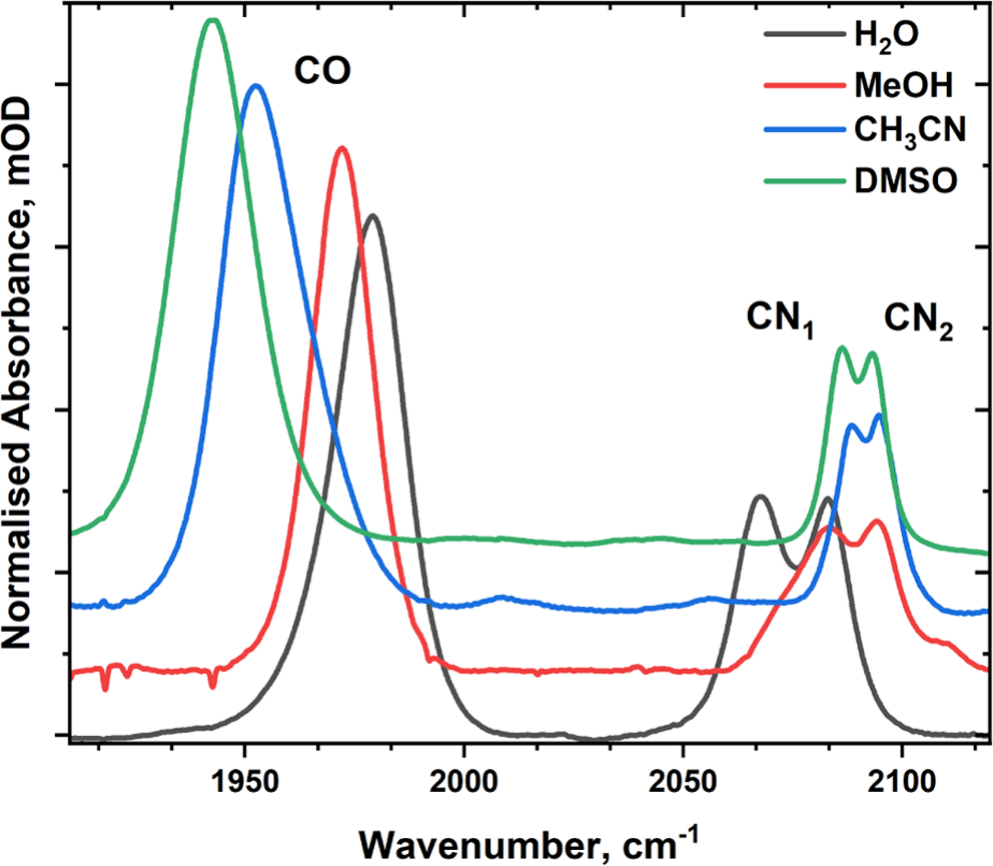
FT-IR spectra of **M1** in H_2_O (black line), MeOH (red), CH_3_CN (blue), and DMSO (green). The corresponding solvent spectrum has been subtracted in all cases. The spectra have been normalized to the ν_CO_ band in H_2_O and vertically offset for clarity. See the SI for the raw spectra.

**M1** has a *C*_s_ symmetry, and there is therefore one a′ CO stretching mode and two CN stretching modes, a′ and a″. The directions of the transition dipoles are in the symmetry plane for a′ and perpendicular to the symmetry plane for a″. In all of the solvents studied, the IR absorption spectrum of **M1** contained three bands in the 1900–2150 cm^–1^ region of the spectrum (Figure 2). A single band in the region 1950–2000 cm^–1^ was assigned to the ν_CO_ mode, and a further two bands in the 2050–2150 cm^–1^ region were assignable to the antisymmetric and symmetric ν_CN_ stretching modes, respectively (labeled CN_1_ and CN_2_). The specific band frequencies are given in Table 1, and the ν_CO_ and ν_CN_ band frequencies observed for **M1** in H_2_O and CH_3_CN solutions agree well with those reported previously, respectively.^37,39^

In solution, the ν_CO_ frequency was observed to increase in more polar protic solvents, following the sequence DMSO, CH_3_CN, MeOH, and H_2_O (the results for D_2_O are identical to those in H_2_O; Table 1). Conversely, the ν_CN_ modes were found to shift to lower frequencies in H_2_O and D_2_O relative to the other three solvents. This leads to a narrowing of the energy gap between ν_CO_ and ν_CN_ bands in the more polar protic solvents. The band separation between ν_CN_ stretching modes was observed to increase from 6 and 8 cm^–1^ in CH_3_CN and DMSO to 11 and 17 cm^–1^ in MeOH and D_2_O/H_2_O, respectively.

The line widths (fwhm) of the ν_CO_ band remained broadly constant (19 ± 3 cm^–1^) in all solvents, while the ν_CN_ bands broadened noticeably from 8 cm^–1^ in DMSO and CH_3_CN to 13 ± 1 cm^–1^ in MeOH and D_2_O/H_2_O.

The spectrum of **M1** as a dry film was more complex than that in solution, with additional ν_CO_ and ν_CN_ bands appearing (see the Supporting Information). The results are in good agreement with previous studies of related complexes where the additional bands were assigned to ν_CO_ and ν_CN_ stretching modes coupled to crystal lattice modes.^37,47−50^ It was established that the measured line widths range from 16 to 22 cm^–1^ for the ν_CO_ modes and from 2 to 5 cm^–1^ for the ν_CN_ modes.

### IR Pump–Probe Spectroscopy

**Figure 3:**
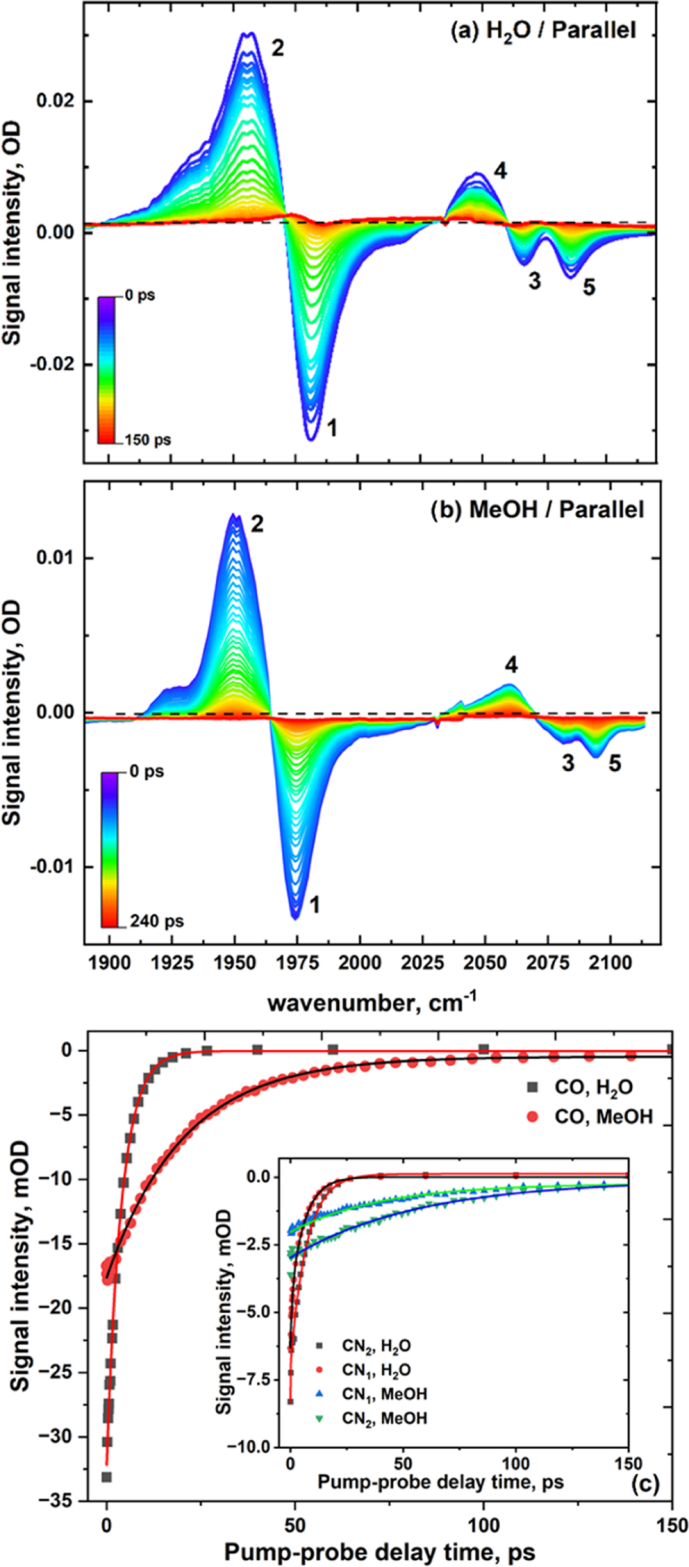
IR pump (2000 cm^–1^)–IR probe spectra of **M1** obtained using parallel pump–probe polarization in (a) H_2_O and (b) MeOH. (c) Time dependence of the amplitudes of peak **1** (ν_CO_, main graph) and peaks **3** and **5** (ν_CN1_ and ν_CN2_, inset) in H_2_O and MeOH. Symbols indicate experimental points, and lines are the best fit to a monoexponential decay function.

IR pump–probe spectra of **M1** in H_2_O and MeOH featured three bands with negative amplitudes alongside positive features (Figure 3a,b). Pump–probe spectra are displayed as pump-on–pump-off difference spectra such that negative features can be assigned to the bleaching (and stimulated emission) of fundamental (*v* = 0–1) transitions following excitation of the sample by the pump pulse. Positive features are similarly attributable to transitions between higher vibrational levels (e.g., *v* = 1–2), which become accessible following excitation by the pump pulse. The exemplar spectra (Figure 3) are typical of those recorded in all solvents (see the SI). Specifically, bands labeled **1** and **2** (Figure 3a,b) are assigned to the *v* = 0–1 and 1–2 transitions of the ν_CO_ mode, respectively. The *v* = 2–3 transition is also visible at lower frequencies and intensity than those of band **2**. The separation of peaks **1** and **2** along the probe frequency axis of the spectrum indicates the anharmonic shift of the *v* = 1–2 transition of the ν_CO_ mode relative to the fundamental transition (*v* = 0–1). Peaks **3**–**5** (Figure 3a,b) arise from ν_CN_ modes. Peaks **3** and **5** are attributable to the *v* = 0–1 transitions of the ν_CN1_ and ν_CN2_ modes, and peak **4** is assignable to an excited vibrational-state transition (*v* = 1–2) of the ν_CN_ modes but cannot be assigned definitively without the detailed knowledge of the relative anharmonic shifts of the ν_CN_ modes, which are revealed by 2D-IR spectroscopy (below).

The amplitudes of all peaks in the IR pump–probe spectra were observed to decay toward the baseline, with an increasing pump–probe time delay in a manner well-represented by a monoexponential decay function (Figure 3c). The time scales of these exponentials under magic angle pump–probe polarization conditions (54.7°) were calculated from the measured parallel and perpendicular data sets (S_magic_ = 1/3 (S_para_ + 2S_perp_)) to reveal the vibrational lifetimes (T_1_) of the *v* = 1 level of the ν_CO_ and ν_CN_ modes (Table 1). The T_1_ value of the ν_CO_ mode was found to be similar in all solvents, covering the range 20 ± 5 ps except in the case of H_2_O, where a value of 5 ± 1 ps was observed; D_2_O gave instead a T_1_ of 24 ± 1 ps. In contrast, the T_1_ values of the ν_CN_ modes varied markedly with the solvent, ranging from 147 ± 14 ps in DMSO to 7 ± 1 ps in H_2_O and generally decreasing with increasing polar and protic nature of the solvent. For **M1** as a dry film, a biexponential decay function was found to produce a better fit result, with T_1_ values of 15–18 (±1) ps and 130–143 (±12) ps being obtained for the ν_CO_ and ν_CN_ modes, respectively. An additional fast component was also observed on the order of 200 ± 100 fs for the ν_CO_ mode and 850 ± 50 fs for the ν_CN_ modes.

In addition to vibrational lifetimes, IR pump–probe data obtained using parallel and perpendicular pump–probe polarization conditions provide access to the anisotropy of the signal (S_aniso_ = (S_para_ – S_perp_)/(S_para_ + 2S_perp_)).^44^ For all of the CO and CN stretching modes studied, a single exponential decay of the anisotropy was observed, with time scales ranging from 4 to 11 ps (ν_CO_) and 0.5 to 7 ps (ν_CN_) (Table S1, see the SI). In general, the time scales were found to be shorter for the ν_CN_ modes than for the ν_CO_ mode in the same solvent. As these time scales are too short to be consistent with molecular reorientation,^51^ we assign this time scale to intramolecular vibrational energy redistribution (IVR) among the ν_CO_ and ν_CN_ modes prior to relaxation to the ground state. We have no way of distinguishing the possible ways by which IVR could take place but acknowledge that this energy redistribution can occur either via the low-frequency modes of M1 or via a solvent-assisted mechanism. We can say, however, that these time scales are qualitatively comparable to the rise time of the cross peaks.

### 2D-IR Spectroscopy

**Figure 4:**
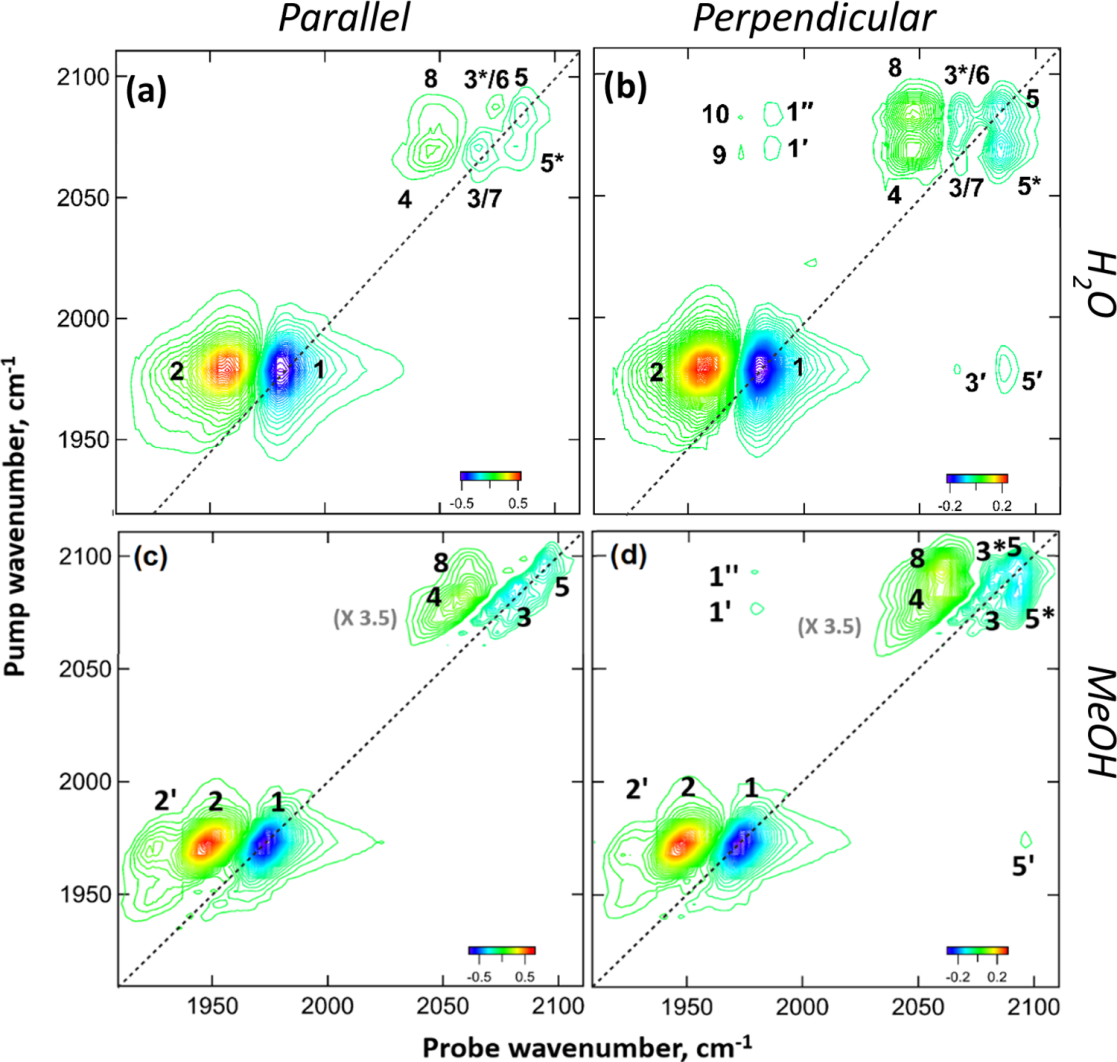
2D-IR spectra of **M1** in H_2_O (a, b) and MeOH (c, d) recorded at a waiting time (*T*_w_) of 800 fs with parallel polarization (a, c) and perpendicular polarization (b, d). Dashed lines indicate the spectrum diagonal. Numbers in parentheses (gray) indicate the magnification of the ν_CN_ region of the spectrum compared to that of ν_CO_.

The 2D-IR spectra of **M1** in H_2_O and MeOH (Figure 4) display several diagonal and off-diagonal peaks. These spectra are representative of those recorded for **M1** in each of the solvents (see the SI), and so the spectrum of **M1** in H_2_O is assigned in detail below and used as a reference point to interpret the results in other solvents.

The 2D-IR spectrum of **M1** can be described as a two-dimensional map of the coupling and energy-transfer patterns between the vibrational modes that give rise to peaks in the IR absorption spectrum. The fundamental (*v* = 0–1) ν_CO_ and ν_CN_ bands visible in the IR absorption spectrum of **M1** appear on the 2D-IR spectrum diagonal along with the respective transient absorptions arising from the *v* = 1–2 transition, with off-diagonal peaks providing additional information on the anharmonicities, vibrational couplings, and energy-transfer pathways.

**Figure 5:**
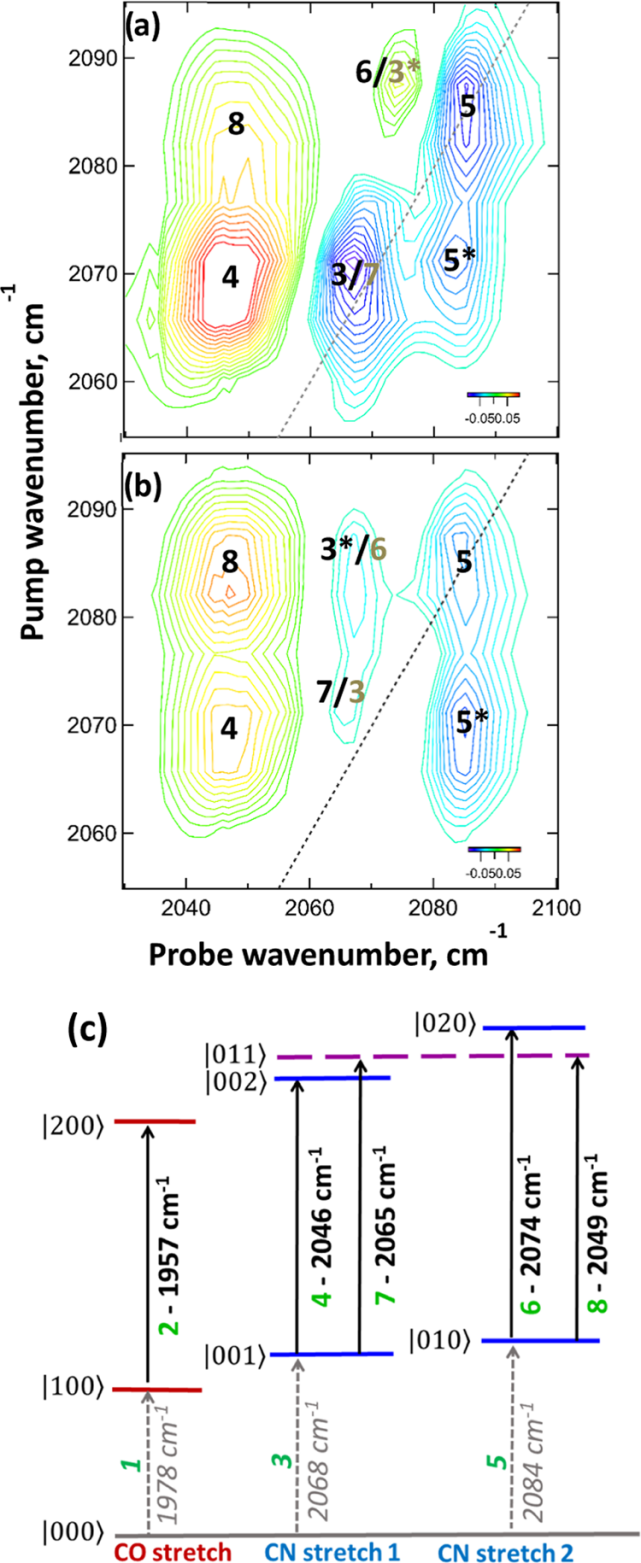
Magnification of the ν_CN_ region of the 2D-IR spectrum of **M1** in H_2_O recorded at a waiting time (*T*_w_) of 800 fs using (a) parallel and (b) perpendicular polarizations. The dashed lines indicate the spectrum diagonal. (c) Energy level diagram showing vibrational energy levels and transition energies of the ν_CO_ and ν_CN_ vibrational manifolds, as detected for **M1** in H_2_O. Transitions are labeled with numbers used to identify peak assignments in the 2D-IR spectra and text. Corresponding frequencies are reported alongside the arrows.

The 2D-IR spectrum of **M1** in H_2_O in the ν_CO_ region (pump and probe frequencies between 1900 and 2000 cm^–1^) obtained by using parallel pump–probe polarization at short values of *T*_w_ (800 fs) contains two peaks (Figure 4a). Peak **1**, a negative peak (blue), lies on the diagonal of the spectrum at (pump, probe) coordinates of (1978, 1978 cm^–1^), while positive (red) peak **2** occurs at the same pump frequency but is shifted by 24 cm^–1^ to a lower probe frequency. These peaks can be assigned in the same manner as the ν_CO_ bands given the same numbers in the pump–probe spectrum (Figure 3a) to the *v* = 0–1 and 1–2 transitions of the ν_CO_ mode of **M1**. The separation of peaks **1** and **2** (24 cm^–1^) provides the anharmonic shift of the ν_CO_ mode (*intra*mode anharmonicity; Table 1). The peak assignments for **M1** in H_2_O are summarized in an energy level diagram shown in Figure 5c, with those for other solvents given in the SI (Figure S9). The equivalent 2D-IR spectrum of **M1** in MeOH (Figure 4c) also shows an additional small peak to the low probe frequency side of **2**, which is assigned to the *v* = 2–3 transition of the ν_CO_ mode. This peak is visible as a result of the slightly narrower line widths observed in MeOH as compared to that of H_2_O (Table 1) and displays the same shift to lower wavenumbers measured for the *v* = 1–2 transition.

In the 2D-IR spectrum of **M1** in H_2_O obtained using perpendicular pump–probe polarization at *T*_w_ = 800 fs (Figure 4b), a pair of negative off-diagonal peaks (**3′** and **5′**) are visible at a pump frequency, which corresponds to the ν_CO_ mode frequency and probe frequencies that match the ν_CN_ bands visible in the IR absorption spectrum. These peaks indicate that the ν_CO_ and ν_CN_ bands of **M1** are vibrationally coupled. In the notation employed, the prime in the peak number (**3′** and **5′**) indicates an off-diagonal peak with a probe frequency matching transition **3** or **5** in the diagram in Figure 5. These weak peaks are accentuated by the perpendicular relative polarization of the pump and probe pulses because of the orthogonal angle between the respective transition dipole moments of the ν_CO_ and ν_CN_ symmetric modes as referenced to the ν_CN_ antisymmetric mode. Detailed analysis of peaks **3′** and **5′** (see the SI) shows that each negative peak is accompanied by a weak positive peak shifted by ∼6 cm^–1^ to a lower probe frequency. These bands are due to a transition between the *v* = 1 level of the pumped ν_CO_ mode and a combination state featuring one quantum of excitation in both the ν_CO_ and the respective ν_CN_ mode. In this case, the frequency shift between the negative and positive peaks indicates the *inter*mode anharmonicity, which provides a measure of the coupling strength of the two modes.^44,52,53^ A value of 6 cm^–1^ indicates that the ν_CO_ and ν_CN_ modes are weakly coupled.

In the ν_CN_ region of the 2D-IR spectrum of **M1** in H_2_O (Figure 4a,b with pump and probe frequencies between 2050 and 2100 cm^–1^), a number of peaks are visible and so this region of the spectrum is expanded for clarity in Figure 5a,b. Two negative peaks are visible on the diagonal of the spectrum obtained under parallel polarization conditions at (pump = probe) 2067 and 2084 cm^–1^ (Figure 5a; **3** and **5**). These are assigned, as in the pump–probe spectrum (Figure 3) to the *v* = 0–1 transitions of the two ν_CN_ modes of **M1**. An off-diagonal peak (5*) below the diagonal link peaks **3** and **5** shows that they are vibrationally coupled. Parallel polarization accentuates transitions with the same transition dipole moment orientation as that of the excited (pumped) mode, and so Figure 5a clearly shows the positions of the two positive *v* = 1–2 transitions (**4** and **6**), which accompany the diagonal peaks (**3** and **5**). The probe frequencies are shown in Figure 5c. From the respective separations of the positive and negative peaks, it can be seen that the anharmonic shift of the higher-frequency ν_CN_ mode (symmetric stretch CN2 in Figure 5c) is much smaller (10 cm^–1^) than that of the lower-frequency mode (antisymmetric stretch CN1; 22 cm^–1^).

Switching to perpendicular polarization (Figure 5b) enhances peaks that arise from interactions between the two ν_CN_ modes, which occurs as a result of the 90° angle between the transition dipole moments of the symmetric and antisymmetric ν_CN_ stretching modes (CN1 and CN2). Thus, in Figure 5b, the negative peak 5* becomes relatively stronger in comparison to the diagonal peaks (**3** and **5**), while two additional features (**7** and **8**) become more clearly visible. Peak **8** indicates the transition from the *v* = 1 level of the CN2 mode to the combination band featuring one quantum of excitation in each ν_CN_ mode. The effects of vibrational coupling shift this mode downward in frequency from the sum of the two *v* = 0–1 fundamental transitions; this is termed the intermode or off-diagonal anharmonicity. From peak **8**, this value was determined to be 19 cm^–1^ for the ν_CN_ mode CN2, indicating considerably stronger coupling between the two ν_CN_ modes than was observed between ν_CO_ and ν_CN_ modes (6 cm^–1^). Peak **7** is the equivalent transition to the combination band following the excitation of CN1. This too displays an intermode anharmonic shift of 19 cm^–1^, which places it very close to the diagonal peak **3**. The overlap results in a partial cancellation of the negative peak **3** and the positive peak **7**, such that the diagonal feature marked **3/7** is significantly weaker under perpendicular polarization conditions (Figure 5b) than under parallel conditions (Figure 5a).

A weak set of off-diagonal features (**1′**, **1”**) arises from the coupling of the ν_CN_ bands to the ν_CO_ band, equivalent to peaks **3′** and **5′** below the diagonal.

Applying similar analyses to spectra of **M1** in all solvents shows that the vibrational coupling of the ν_CO_ and ν_CN_ modes and between the ν_CN_ modes is not sensitive to the solvent. Comparing the values of the intramode anharmonic shift for the ν_CO_ and ν_CN_ modes (Table 1) also shows that the value for the ν_CO_ mode is virtually insensitive to the identity of the solvent (24–25 cm^–1^), while the anharmonic shift of the low-frequency ν_CN_ (CN1) mode shows a weak variation from 19 cm^–1^ in MeOH to 22–23 cm^–1^ in H_2_O/D_2_O, DMSO, and CH_3_CN. By contrast, the anharmonic shift of CN2 varies strongly with the solvent, increasing by more than a factor of 2 from 10 cm^–1^ in H_2_O and D_2_O to 20 cm^–1^ in DMSO and CH_3_CN, with MeOH producing an intermediate value of 16 cm^–1^. In the dry film, the ν_CO_ and low-frequency ν_CN_ (CN1) modes were both found to have intramode anharmonic shifts of 17 cm^–1^, while for CN2, a very low value of 2 cm^–1^ was observed.

**Figure 6:**
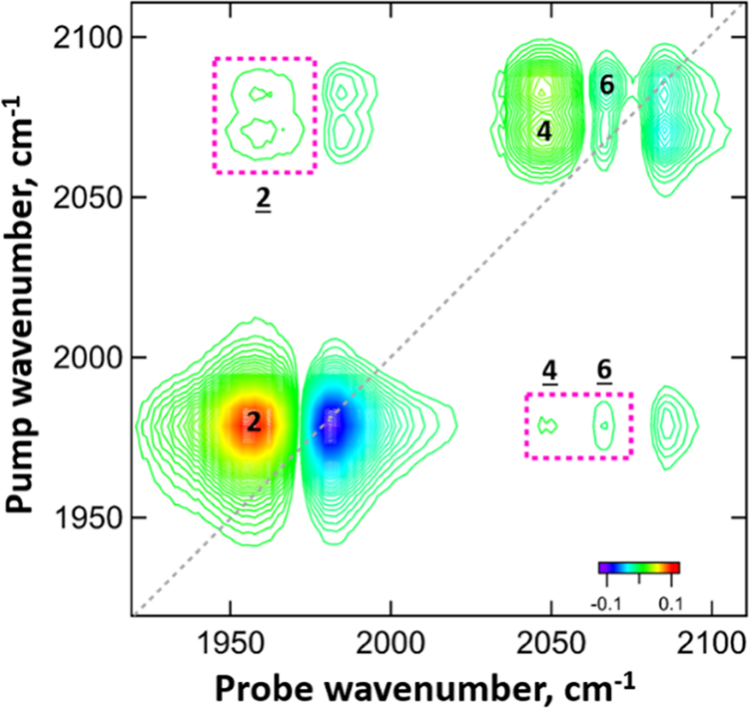
2D-IR spectra of **M1** in H_2_O recorded at a waiting time (*T*_w_) of 5 ps in perpendicular polarization. The dashed line indicates the spectrum diagonal. The underlined numbers refer to energy-transfer peaks, which are also highlighted by the dashed rectangles.

Analysis of the spectra at long T_W_ (≥5 ps) allows the identification of new peaks arising from energy transfer between the different modes, which support the peak assignment presented above. These peaks arise as a consequence of the vibrational energy redistribution from the *v* = 1 state of the excited (pumped) mode to the *v* = 1 level of modes lying at neighboring frequencies. The probe pulse is then able to excite the *v* = 1–2 transition of the indirectly populated state, which will appear as a new peak at a time scale consistent with the relaxation dynamics of the pumped mode. Analysis of our spectra shows the appearance of these features in all of the investigated solvents. In fact, energy is transferred to the CN modes when pumping the CO and *vice versa*. Determination of the energy redistribution between the CN modes is less clear as the peaks overlap. This concept is exemplified by analyzing the behavior of **M1** in H_2_O at a *T*_w_ of 5 ps (Figure 6). Peak 2 indicates energy transfer from the *v* = 1 state of the excited (pumped) ν_CN1_ or ν_CN2_ mode at 2068 and 2084 cm^–1^, respectively, to the *v* = 1 level of the ν_CO_ mode at 1978 cm^–1^. The probe pulse excites the *v* = 1–2 transition of the 1978 cm^–1^ mode, which lies at 1957 cm^–1^. The appearance of peaks **2** is therefore due to energy transfer between the ν_CN_ modes and the CO mode. The reverse peaks featuring energy transfer from the pumped CO mode at 1978 cm^–1^ to the 2068 and 2084 cm^–1^ modes followed by probe excitation of the *v* = 1–2 transition of the CN modes are indicated by peaks **4** and **6**, respectively.

### 2D-IR Structural Dynamics

The structural dynamics exhibited by the ν_CO_ and ν_CN_ vibrational modes can be measured via 2D-IR spectroscopy by analysis of the variations in the 2D-line shape of the diagonal (*v* = 0–1) peaks with *T*_w_. In solution, the ν_CO_ and ν_CN_ bands of **M1** become inhomogeneously broadened as a result of the range of microenvironments experienced by the solute due to solvent motion or H-bond exchange. In a 2D-IR spectrum, at shorter values of *T*_w_, an inhomogeneously broadened band exhibits elongation along the diagonal of the spectrum, while at longer *T*_w_, the ensemble evolves between the pump and probe events leading to a more circular line shape. This phenomenon has been well documented elsewhere.^44^ The process of line shape evolution, referred to as spectral diffusion, can be quantified by a range of equivalent methods.^44^ Here, we apply the nodal line slope (NLS) approach in which the inverse of the gradient of the node between the negative (*v* = 0–1) and positive (*v* = 1–2) peaks near the spectrum diagonal is plotted as a function of *T*_w_, providing a measure of the local dynamics experienced by the pumped vibrational mode.^44,54^

**Figure 7:**
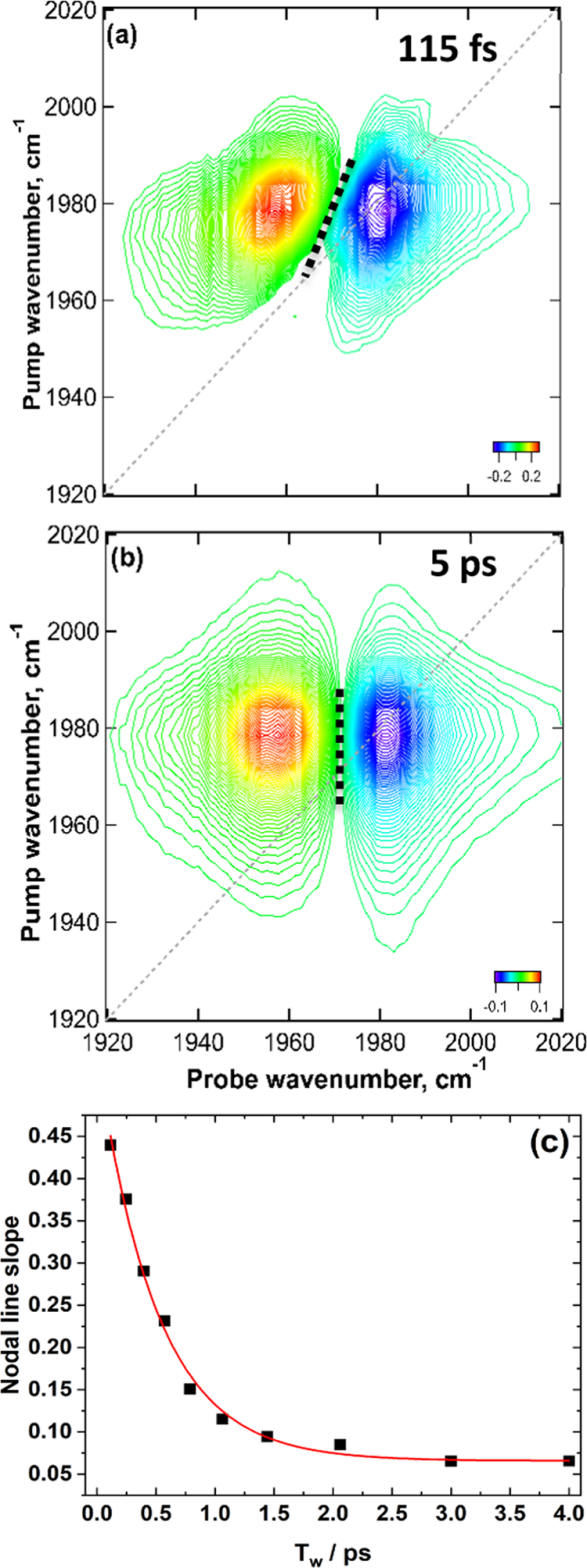
2D-IR spectra of **M1** in H_2_O recorded at a waiting time (*T*_w_) of 115 fs (a) and 5 ps (b) in the ν_CO_ region. The dotted line indicates a nodal line. (c) Temporal dependence of the nodal line slope to obtain a qualitative measure of the frequency fluctuation correlation function. The red line is a monoexponential fit to the experimental data.

The results of applying the NLS analysis to the ν_CO_ mode of **M1** in H_2_O are shown in Figure 7a–c. At a *T*_w_ value close to zero (Figure 7a), the nodal line shows a marked tilt toward the diagonal (large NLS; Figure 7c), which evolves until the nodal line is near vertical (low NLS) at longer *T*_w_ (Figure 7b,c). Plotting the NLS value over a range of *T*_w_ values produces a decay, which could be well-represented by a single exponential function (Figure 7c) with a time scale of 0.5 ps.

The spectral diffusion time scales for the ν_CO_ mode of **M1** in other solvents (Table 1) show a range of values from 0.5 ± 0.1 ps in H_2_O to 1.6 ± 0.1 ps in DMSO and 2.1 ± 0.5 ps in MeOH (Table S2, see the SI).

## Discussion

The new experiments on **M1** provide a comprehensive picture of the vibrational spectra, couplings, and energy-transfer pathways within the [Fe(CO)(CN)_2_] unit and yield a framework to compare quantitatively with analogous data determined previously from [NiFe] hydrogenases, therefore affording further insights into the nature of the active site and its environment.

The IR absorption spectra reported compare favorably with previous work, which concluded that the reduction in ν_CN_ and an increase in ν_CO_ fundamental frequencies upon changing from aprotic to protic media are attributable to changes relating to the solvation of the K^+^ counterion.^37^ In DMSO and CH_3_CN, contact counterion pairing occurs between K^+^ and the anionic CN ligands, whereas in water (and D_2_O), the K^+^ ion is fully solvated, leading to H-bonds forming between water and the CN ligands. It was concluded based on the good agreement between the spectra of **M1** in CH_3_CN and that of the enzyme from *C. vinsoum* that the active site pocket offered interactions with the protein more reminiscent of contact ion pairing than with the optimum H-bonding offered by bulk water.^37^ Our IR absorption data for **M1** agree well with this previous study, though comparisons with *Cn*RH and *Ec*Hyd1, for which ultrafast spectroscopy data exist (Table 1), provide less clear-cut agreement with the spectroscopy of **M1**. While the low ν_CO_ frequency observed for each enzyme is more akin to aprotic solvents, the ν_CN_ frequencies are not clearly identifiable with the values for protic and aprotic media. The band frequencies are, however, state-dependent,^55^ and a wider survey of the literature shows that the ν_CN_ bands of the more oxidized states of most [NiFe] hydrogenases reported show better agreement with **M1** in aprotic media than protic solvents.^1,13,56^ The ν_CN_ values obtained for **M1** in the dry film also show good agreement with aprotic solvents, as expected given that ion pairing would be anticipated in the solid phase.^39^

The results of the IR pump–probe spectroscopy measurements provide information on the vibrational relaxation times of the ν_CO_ and ν_CN_ modes of **M1**. Relaxation from higher vibrational levels proceeds via either an intramolecular mechanism or direct mediation by the solvent, meaning that the values determined from **M1** provide a useful benchmark for comparison with enzyme data. Table 1 shows that the ν_CO_ vibrational lifetime is rather insensitive to changes in its environment, with the value varying by only a few picoseconds from D_2_O to DMSO and the dry film: the observed lifetimes, on the order of 20 ps, agree well with those from the hydrogenases *Cn*RH and *Ec*Hyd1. This insensitivity suggests that an intramolecular relaxation route may well dominate ν_CO_ relaxation. The one notable exception to this is **M1** in H_2_O, where a significant acceleration of vibrational relaxation occurs (T_1_ = 5 ps). This increased relaxation rate is assignable, via previous work on water-soluble organometallic species,^24^ to the effect of an energy overlap between a combination band of the water H–O–H bend and librational (δ_H–O–H+libr_) modes near 2100 cm^–1^ with the ν_CO_ stretching modes of organometallic carbonyls. This resonance provides an efficient route to vibrational relaxation when bulk water is present and shows that an intermolecular relaxation route via the solvent exists for the ν_CO_ modes, but this apparently rarely outcompetes the intramolecular relaxation route. As this fast relaxation is not replicated for ν_CO_ modes in the enzymes, this supports the previously stated conclusion that the active site is free of bulk water.^20,22^

In the case of the ν_CN_ modes, the strong solvent dependence of the T_1_ relaxation time shows that an intermolecular relaxation mechanism dominates. Fast relaxation is present in H_2_O, as observed for the ν_CO_ modes and can be assigned to the same resonance effect with the δ_H–O–H+libr_ band. Removal of this resonance, as in D_2_O, slows down the relaxation rate dramatically, showing that fast relaxation is not simply a result of H-bonding or protic solvents. In the solvents where counterion pairing dominates, the slow relaxation suggests that ion-paired K^+^ inhibits relaxation through the solvent. The good agreement between the ν_CN_ lifetimes of **M1** in DMSO and the data from the dry film is noteworthy, however, and could indicate that the ∼140 ps time scales observed are assignable to an intramolecular relaxation route or that relaxation rates through the solid-state matrix and DMSO are similar.

In the case of the ν_CN_ modes, the sensitivity of the T_1_ time scales offers a good point of comparison for data obtained from the enzyme, where values of 30–40 ps (*Ec*Hyd1) and 30 ps (*Cn*RH) compare well with **M1** in D_2_O and MeOH, indicating that the CN ligands interact with the protein scaffold in a manner similar to a protic, though not bulk aqueous, solvent. This is consistent with the conclusions drawn previously via IR absorption studies on **M1** in that the enzyme environment does not resemble the idealized H-bonding in bulk water,^37^ though the new relaxation time data add a little more information, leading toward a more protic, organic solvent-like environment.

The results of 2D-IR spectroscopy measurements on **M1** show many similarities to measurements on the enzymes. The weak coupling observed between ν_CO_ and ν_CN_ modes (6 cm^–1^) and the strong (19 cm^–1^) coupling between ν_CN_ modes are not solvent-dependent and are very close to values reported for *Ec*Hyd1 and *Cn*RH.^20,22^ They also agree well with studies using isotopic labeling and IR absorption spectroscopy^37,39^ and with DFT calculations using the [NiFe] site including only the first coordination sphere.^57^ This agreement extends to the anharmonic shifts of the vibrational bands associated with the ν_CO_ and ν_CN_ vibrational coordinates. The intramode anharmonic shifts for the ν_CO_ mode of **M1** and the low-frequency ν_CN_ mode (CN1) show little solvent dependence and agree well with the values obtained for *Ec*Hyd1 and *Cn*RH.^20,22^

A significant deviation is, however, observed for the intramode anharmonic shift of the higher-frequency ν_CN_ mode (CN2). For this mode, the anharmonic shift ranged from 2 cm^–1^ in the crystalline phase of **M1** to 8 cm^–1^ in *Ec*Hyd1 and *Cn*RH, 10 cm^–1^ in H_2_O/D_2_O of **M1**, 16 cm^–1^ in MeOH, and 20 cm^–1^ in CH_3_CN and DMSO. This shows that the anharmonicity of the ν_CN_ mode CN2 is a sensitive reporter of its local environment. Studies of anharmonicities of organometallic cyanide species in solution are rare but values tend toward the higher values observed for CN1,^25,58^ suggesting that the low CN2 value is specific to the solid state, enzyme, or H_2_O-like environments containing two CN ligands and one carbonyl. Ultrafast IR studies (pump–probe and 2D-IR) on CpFe(CO)_2_(CN) (see the SI) yielded a value for an intramode anharmonicity of 15 cm^–1^ for both the ν_CO_ modes and 24 cm^–1^ for the ν_CN_ mode.

A recent DFT study^57^ successfully reproduced the anharmonic shifts of the two CN modes and concluded that the difference in intramode anharmonicities is due to an intrinsic, first coordination sphere, feature of an isolated Fe(CO)(CN)_2_ unit, arising from a 2–2 Darling–Dennison resonance involving the second excited states of the v_CN_ modes of the Fe(CO)(CN)_2_ moiety.^57^ Our new data suggest that the situation may be more nuanced. If such a resonance results in the disparate anharmonic shifts of the two ν_CN_ modes, as proposed, then our data indicate that the extent of the resonance overlap and coupling is very sensitive to, or tuned by, the local molecular environment. The dry film, which precludes the bulk solvent, produces a very low intramode anharmonicity. One interpretation is that the solid-state environment of **M1** gives insight into the behavior of the isolated molecule; however, this must be applied with caution as changes to the IR absorption spectrum of **M1** in the dry film indicate that unique modes arise from the crystal lattice. These additional bands could therefore indicate the presence of strong inter-**M1** interactions not found in solution or possibly be the result of very strong counterion pairing effects. In the presence of strong H-bonding to **M1** in aqueous solution, a situation similar to that of the enzyme arises, while other solvents do not produce this effect. This leads to the conclusion that, while the first coordination sphere models show that the disparity in ν_CN_ mode anharmonicities may well be an intrinsic characteristic, the protein scaffold also plays an important role in modulating the Fe(CO)(CN)_2_ unit and is responsible for the magnitude of the observed effect. It is noteworthy that all other parameters reported comparing **M1** to enzymes have produced values suggesting that the enzyme active site most closely resembles MeOH or a non-H_2_O protic solvent, involving weak H-bonding to the CN ligands, suggesting that the H-bond partners in the enzyme are maintained. While the intermode anharmonicity of CN2 does not show perfect agreement with the data for **M1** in MeOH, being closer to that in D_2_O, this is broadly consistent with our findings.

The final observation from 2D-IR spectroscopy relates to spectral diffusion, where all solvents produced fast line shape evolution, indicative of a dynamic local environment. This was replicated even for the ν_CO_ modes, which do not seem to interact strongly with their environment but clearly sense the local dynamics, a process that may be mediated by coupling between CN and CO ligand vibrational modes. The observed fast spectral diffusion is in marked contrast to enzyme measurements, which show virtually no spectral diffusion and little inhomogeneous broadening consistent with all previous observations indicating that the enzyme creates a very constrained second coordination sphere for the [NiFe] center with little inhomogeneity or structural fluctuation on picosecond time scales. The same scenario is observed for the dry film of **M1** where the picosecond time scale spectral diffusion is absent.

Taken together, the clear indication from our data is that the presence or absence of H-bonds with the CN ligands is instrumental in defining many of the characteristics of **M1** in solution. By way of confirmation, a series of studies were conducted using IR absorption and IR pump–probe spectroscopy of **M1** in H_2_O/DMSO mixtures with different solvent ratios (see the SI). The results clearly show that rather than the change from H_2_O to DMSO-like behavior occurring in a linear fashion, the transition begins only after a ratio of 40% (by molecule) H_2_O is reached. Vibrational frequencies are shifted by 20 cm^–1^ for the CO and CN1 modes from neat DMSO to an 80% water/DMSO mixture; less accentuated is the shift observed for the CN2 mode (10 cm^–1^) under the same experimental conditions. Pump–probe data collected for **M1** in a mixture of 70–80% water versus DMSO displayed accelerated lifetimes compared to neat DMSO (10 ± 1 ps for the CO mode and between 14 and 19 (±5) ps for the CN modes, rendering these values akin to those observed in aqueous solution) (Table 1). This correlates perfectly with recent studies showing that the DMSO:water mixture exists in three concentration regimes.^59^ Below 30 mol % water, strong interactions between water and DMSO clusters strictly limit the amount of “free” water, but at higher water levels, the solution becomes more ideal. We suggest that this correlation stems from the onset of H-bonding phenomena with **M1** once the water content of the solutions is sufficient to overcome the DMSO clusters and as such this observation adds weight to the assignment of the spectroscopic and dynamic effects observed here to the presence or absence of H-bonding interactions with CN ligands.

The overall picture arising from our study is one in which the CO ligands of the Fe(CO)(CN)_2_ unit do not interact strongly with their environment, either in the solution phase or in the enzyme active site, as demonstrated by the solvent insensitivity of many of the parameters reported. In contrast, the CN ligands provide a point of contact with the local environment, which occurs by virtue of their charge through H-bonds in the enzyme and for **M1** in H_2_O and MeOH. In the case of **M1** in solution, H-bonds are replaced by a counterion pairing mechanism, which can either mediate solvent interaction, as in CH_3_CN, or lead to a quite limited solvent interaction in more extreme cases such as DMSO.^37^ This all supports the conclusion that computational models of the active site of the [NiFe] enzymes would be improved by the inclusion of at least explicit H-bonding interactions.^60−65^ Overall, our results strengthen the belief, also suggested by other studies, that the protein scaffold acts to create a specific molecular environment for the [NiFe] center.^20^ The H-bonds that are central to this behavior have the ability to create a diverse continuum of interactions, which we show clearly through the modulation of the anharmonic shift of the high-frequency ν_CN_ mode by the solvent environment. Further studies to identify the precise means by which this modulation occurs, through seeking to better understand the role of vibronic interactions of the molecule and solvent modes involved in resonance, will be valuable in developing knowledge on the nature of the interaction between protein and catalytic center.

## Conclusions

In this study, IR pump–probe and 2D-IR spectroscopy have been applied to investigate the spectroscopy and vibrational dynamics of the organometallic compound K[CpFe(CO)(CN)_2_] (**M1**) in a number of protic and polar solvents and in the crystalline phase. Measurements of anharmonicity, intermode coupling strength, vibrational relaxation time, and solvation dynamics of the CO and CN stretching modes of **M1** in H_2_O, D_2_O, methanol, dimethyl sulfoxide, and acetonitrile reveal that the presence or absence of H-bonding to the CN ligands plays an important role in defining the fundamental mode frequencies, anharmonicities, vibrational relaxation times, and structural dynamics of ν_CN_ bands, while the ν_CO_ modes interact with the local molecular environment to a lesser degree. Using these data to provide insight into the role of the protein scaffold points to the importance of the H-bonds between nearby amino acid residue side chains and the CN ligands of the [NiFe] center. Alongside recent work showing that anharmonic effects are required in order for quantum computational models to accurately reproduce spectroscopic parameters and potential energy surfaces of the catalytic center,^57^ we believe this work also motivates the inclusion of at least an explicit second coordination sphere. Adding our outcomes to previous studies shows that the molecular dynamics found in solution, while fundamental, highlight the need to develop better experimental models of the actual enzyme for furthering our understanding of the unique nature of the active site created by the protein scaffold and the potential importance of mimicking it accurately in a biomimetic system.

## Supporting Information Available

Negative ESI mass spectrum of K[CpFe(CO)(CN)_2_] (Figure S1); ^1^H-^13^C HMQC of **M1** (Figure S2); FT-IR spectra of **M1** (Figure S3); FT-IR and IR pump-IR probe spectra of **M1** as a dry film (Figure S4); 2D-IR spectra and slices of **M1** as a dry film (Figure S5); IR pump-IR probe spectra of **M1** in parallel polarization (Figure S6); slices through the 2D-IR spectrum of **M1** (Figure S7); 2D-IR spectra of **M1** (Figure S8); energy level diagrams of **M1** (Figure S9); expansion of the v_CO_–v_CN_ cross-peak region of a 2D-IR spectrum (Figure S10); FT-IR, IR pump-IR probe, 2D-IR spectra of CpFe(CO)_2_(CN) (Figure S11); frequency shifts for the CO, CN1, and CN2 modes of **M1** (Figure S12); temporal dependence of the nodal line slope between the 0–1 and 1–2 transitions (Figure S13); anisotropy decays for the CO and CN modes in the different solvents (Figure S14); v_CO_–v_CN_ mode IR frequencies for CpFe(CO)_2_(CN) (Table S1); v_CO_–v_CN_ mode IR frequencies for **M1** (Table S2); and spectral diffusion constants (Table S3) (PDF)
