# Supplementary material for: Understanding the [NiFe] Hydrogenase Active Site Environment through Ultrafast Infrared and 2D-IR Spectroscopy of the Subsite Analogue K[CpFe(CO)(CN)2] in Polar and Protic Solvents
Source: J Phys Chem B. 2024 Feb 1;128(6):1461–72. doi: 10.1021/acs.jpcb.3c07965 (PMC10875664; doi:10.1021/acs.jpcb.3c07965)
Supplement: Supplementary file 1 — jp3c07965_si_001.pdf [file jp3c07965_si_001.pdf]

Understanding the [NiFe] Hydrogenase Active Site Environment Through Ultrafast Infrared and 2D-IR Spectroscopy of the Subsite Analogue $K[CpFe(CO)(CN)_2]$ in Polar and Protic Solvents

Barbara Procacci,[1] Solomon L.D. Wrathall,[1] Amy L. Farmer,[1] Daniel J. Shaw, [1] Gregory M. Greetham,[2] Anthony W. Parker,[2] Yvonne Rippers,[3] Marius Horch,[3] Jason M. Lynam,[1] Neil T. Hunt*[1]

1) Department of Chemistry and York Biomedical Research Institute, University of York, York, YO10 5DD, UK
2) STFC Central Laser Facility, Research Complex at Harwell, Rutherford Appleton Laboratory, Harwell Campus, Didcot, OX11 0QX, UK
3) Freie Universität Berlin, Department of Physics, Ultrafast Dynamics in Catalysis, Arnimallee 14, 14195 Berlin, Germany

Table of contents

Figure S1. Negative ESI mass spectrum of $K[CpFe(CO)(CN)_2]$, **M1** in CH_3CN. S3

Figure S2. ^1H-^{13}C HMQC of **M1** in D_2O. S3

Figure S3. FT-IR spectra of **M1** in (a) H_2O; (b) MeOH; c) CH_3CN: d) DMSO; e) D_2O. S4

Figure S4. FT-IR and IR Pump-IR Probe spectra of **M1** as a dry film. S5

Figure S5. 2DIR spectra and slices of **M1** as a dry film. S5

Figure S6. IR Pump-IR probe spectra of **M1** in parallel polarization in a) D2O; b) CH3CN; c) DMSO. S6

Figure S7. Slices through the 2D-IR spectrum of **M1** in H_2O (top) and MeOH (bottom) under parallel (a) and perpendicular (b) polarization conditions. S7

Figure S8. 2D-IR spectra of **M1** at a T_w of 800 fs in parallel pump-probe polarization in a) D_2O; c) CH_3CN; e) DMSO and perpendicular polarization b) D_2O; d) CH_3CN; f) DMSO. S8

Figure S9. Energy level diagrams of **M1** in MeOH, CH_3CN and DMSO.

Figure S10. Expansion of the ν_{CO} - ν_{CN} cross peak region of a 2D-IR spectrum for **M1** in H_2O. S10

Figure S11. FT-IR, IR Pump-IR Probe, 2D-IR spectra of $CpFe(CO)_2(CN)$ in CH_3CN. S10

Table S1. ν_{CO} and ν_{CN} modes IR frequencies for $CpFe(CO)_2(CN)$ in CH_3CN, anharmonicities and vibrational lifetimes. S11

Table S2. ν_{CO} and ν_{CN} modes IR frequencies for **M1** in mixtures of DMSO/H_2O obtained from Gaussian fitting. S11

Figure S12. Frequency shifts for the CO, CN1 and CN2 modes of **M1** with increasing % of water molecules *versus* DMSO molecules. S12

Figure S13. Temporal dependence of the nodal line slope between the 0-1 and 1-2 transition for the ν_{CO} to obtain a qualitative measure of the frequency fluctuation correlation function in the different solvents. S13

Table S3. Spectral diffusion constants (ps) for ν_{CO} of **M1** obtained in the different solvent by the nodal line slope method.

Figure S14. Anisotropy decays for the CO and CN modes in the different solvents. S13

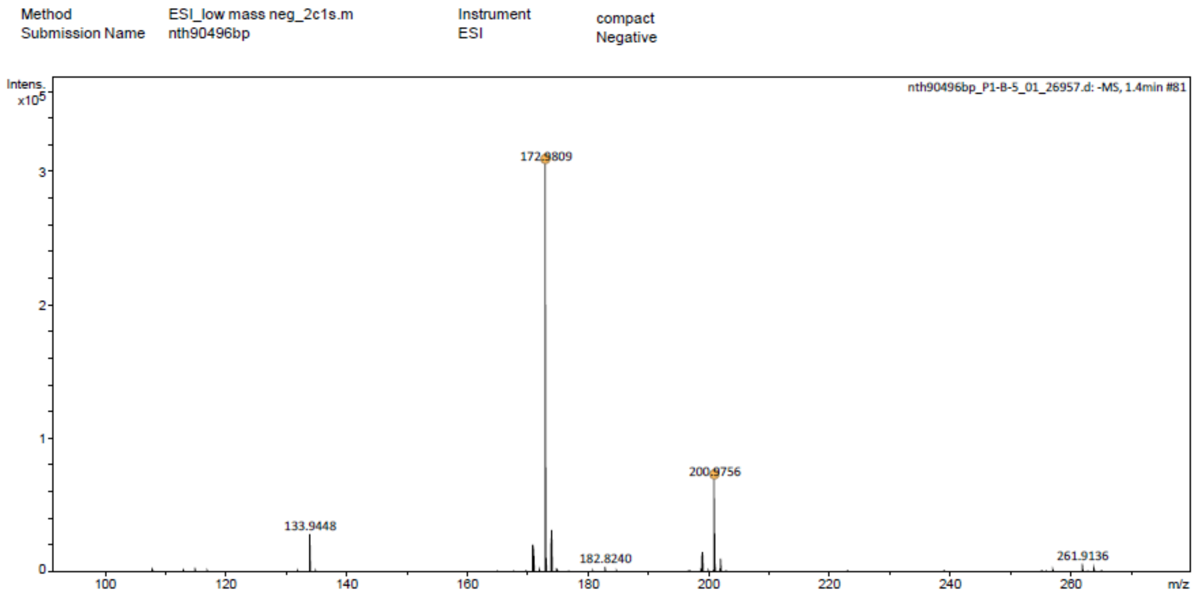

Meas. m/z	#	Ion Formula	m/z	err [ppm]	err [mDa]	mSigma	Mean err [ppm]
172.9809	1	C7H5FeN2	172.9808	-0.7	-0.1	4.8	-0.0
200.9756	1	C8H5FeN2O	200.9757	0.5	0.1	69.8	-0.8

Figure S1. *Negative ESI mass spectrum of* ***M1*** *in CH_3CN showing the peak for the anion $[CpFe(CO)(CN)_2]^-$ (exp m/z = 200.9756; calculated m/z for $C_8H_5FeN_2O$ = 200.9757; diff = 0.1 mDa) and for its fragment after dissociating CO (exp m/z = 172.9809; calculated m/z for $C_7H_5FeN_2$ = 172.9808; diff = -0.1 mDa).*

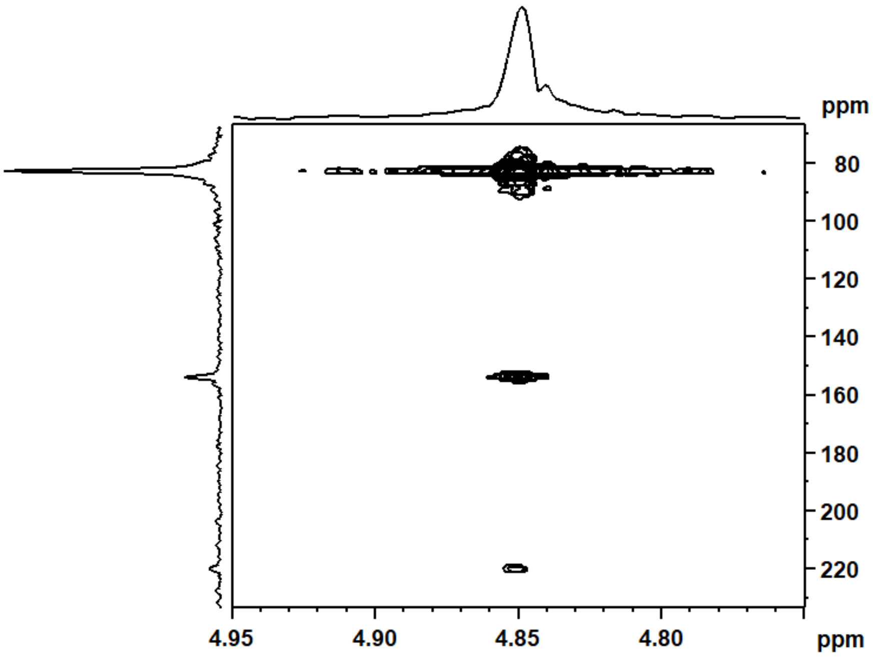

Figure S2. *^1H-^{13}C HMQC of* ***M1*** *in D_2O displaying cross peaks between the resonance for the C_5H_5 (Cp) ligand in the ^1H spectrum and the corresponding carbon peak in the ^{13}C spectrum as well as cross peaks to the CO and the CN ligands.*

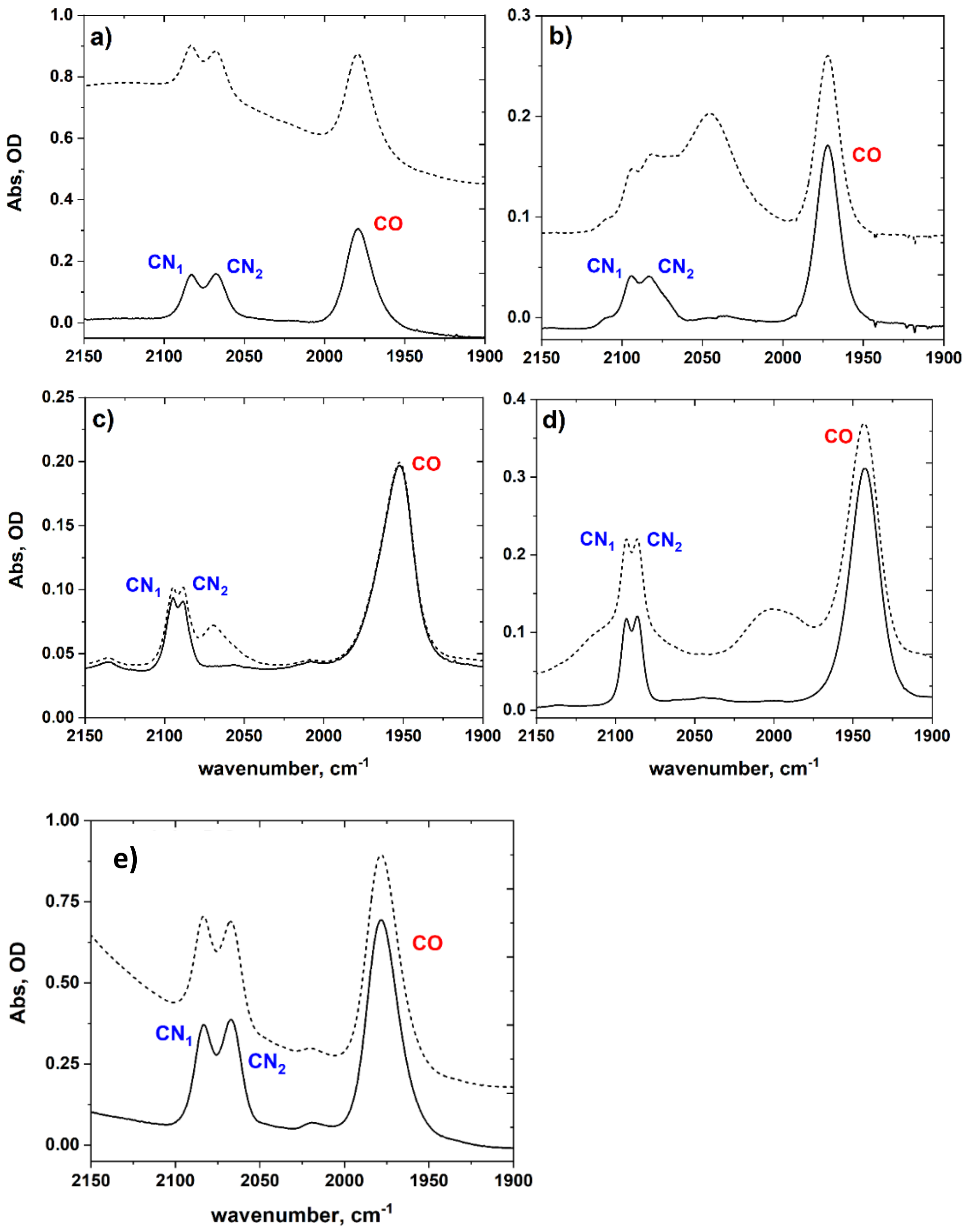

Figure S3. *FT-IR spectra of* ***M1*** *in (a) H_2O; (b) MeOH; c) CH_3CN: d) DMSO; e) D_2O. Dashedand solid lines show spectra before and after solvent subtraction respectively.*

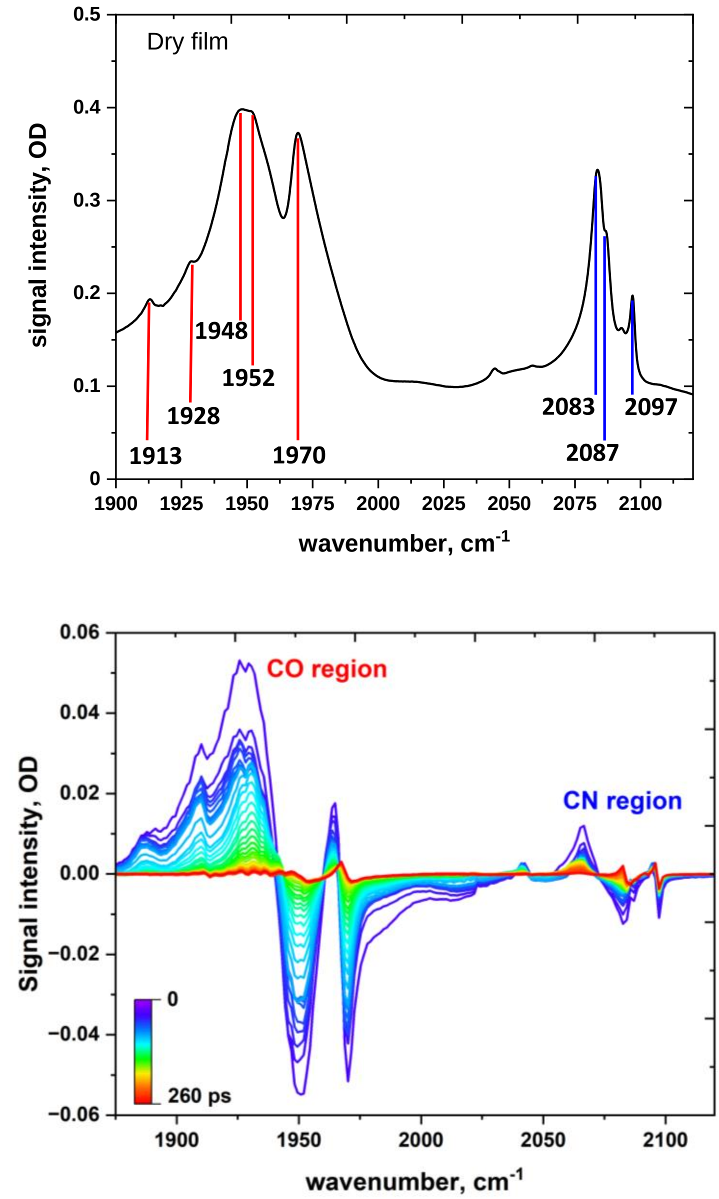

Figure S4. *FT-IR, and IR Pump- IR Probe spectra of **M1** as a dry film. The film was obtained by depositing a CH_3CN solution of **M1** (2.5 mM) on a CaF_2 window and allowing the solvent to slowly evaporate.*

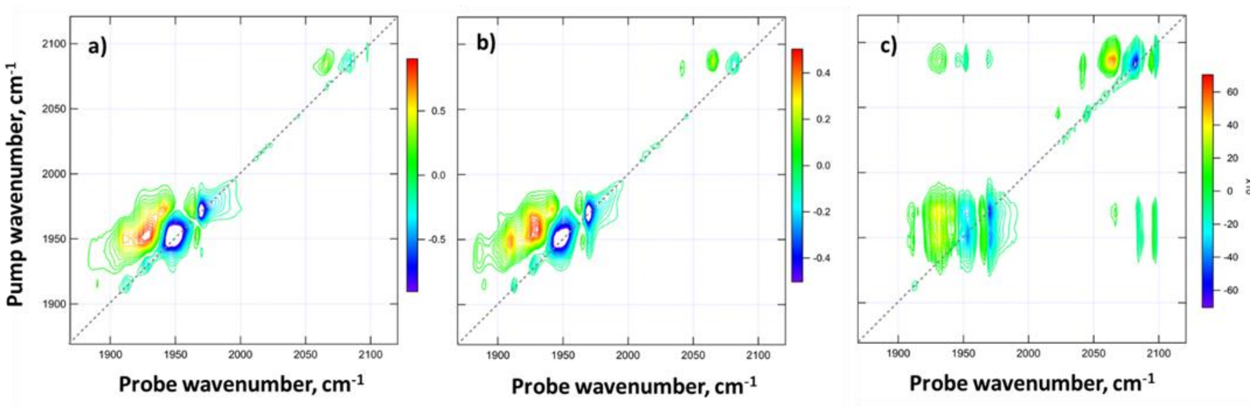

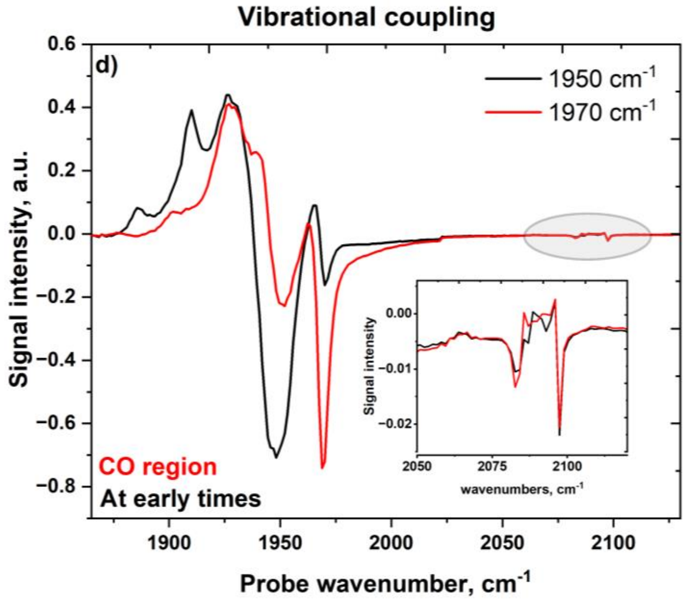

Figure S5. *Top: 2D-IR spectra of **M1** as a dry film at a Tw values of a) 250 fs; b) 1.5 ps; c) 40 ps using parallel polarization. Energy tansfer peaks appear at late delay times as shown in spectrum c). Bottom: Slices through the 2D-IR spectrum under parallel polarization conditions at Tw value of 250 fs. The pump frequency is given in the legend. The inset shows a magnification of the ν_{CN} region to demonstrate cross peaks to the ν_{CO} modes indicating vibrational coupling.*

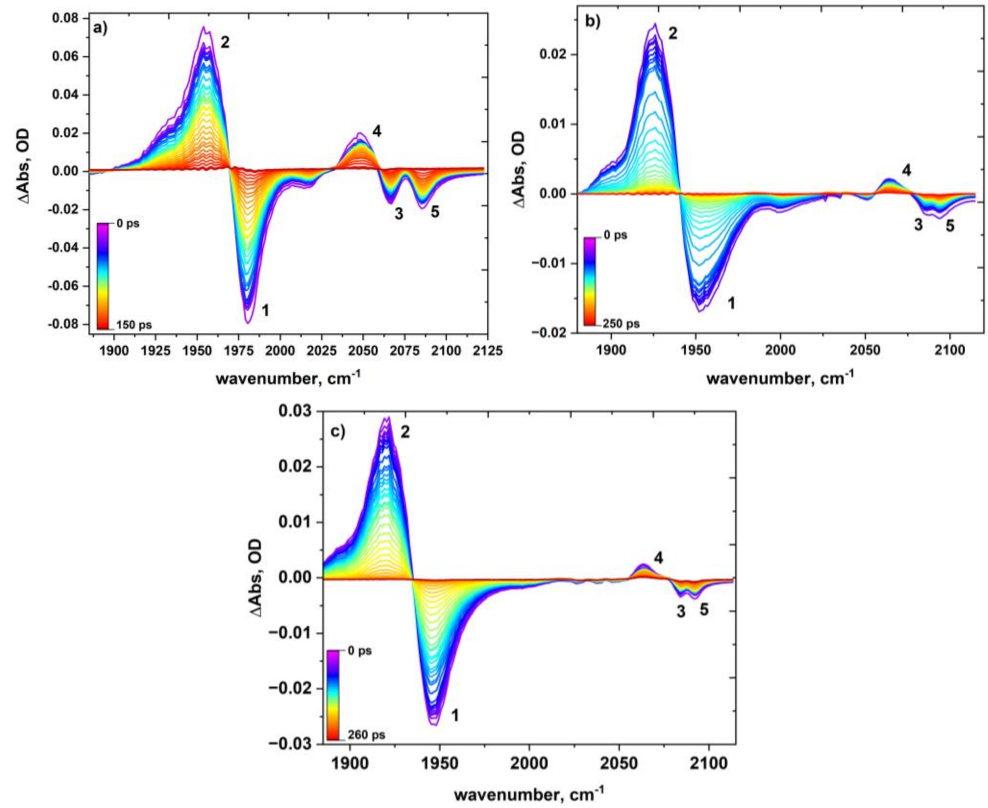

Figure S6. *IR Pump-IR probe spectra of **M1** using parallel polarization in a) D_2O; b) CH_3CN; c) DMSO.*

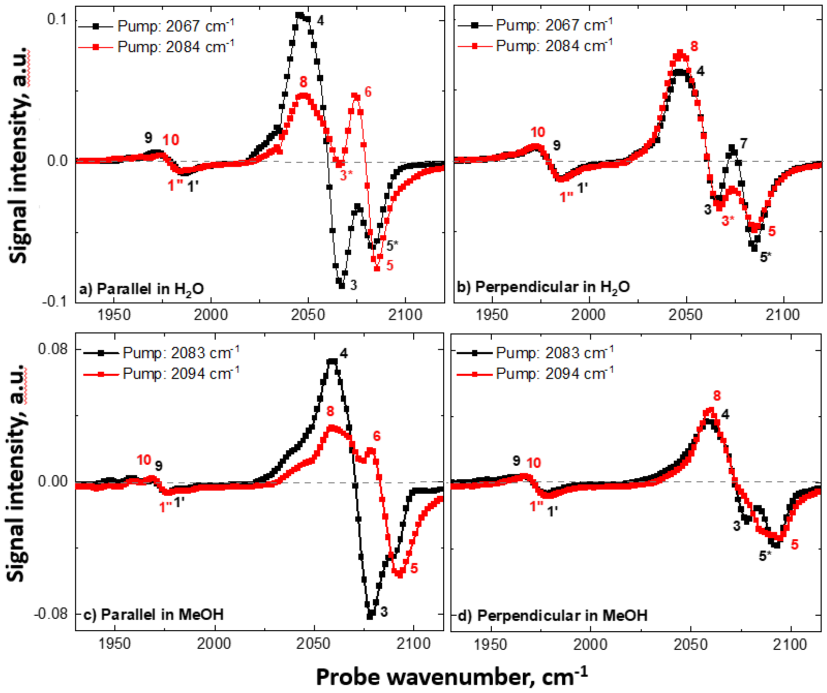

Figure S7. *Slices through the 2D-IR spectrum in H_2O (top) and MeOH (bottom) under parallel (a) and perpendicular (b) polarization conditions. The pump frequency in each case is given in the legend. Numbers correspond to those used in the main text.*

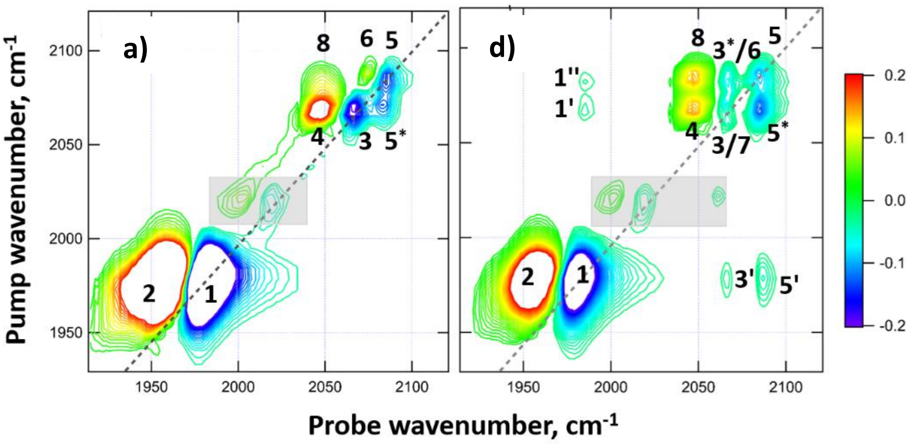

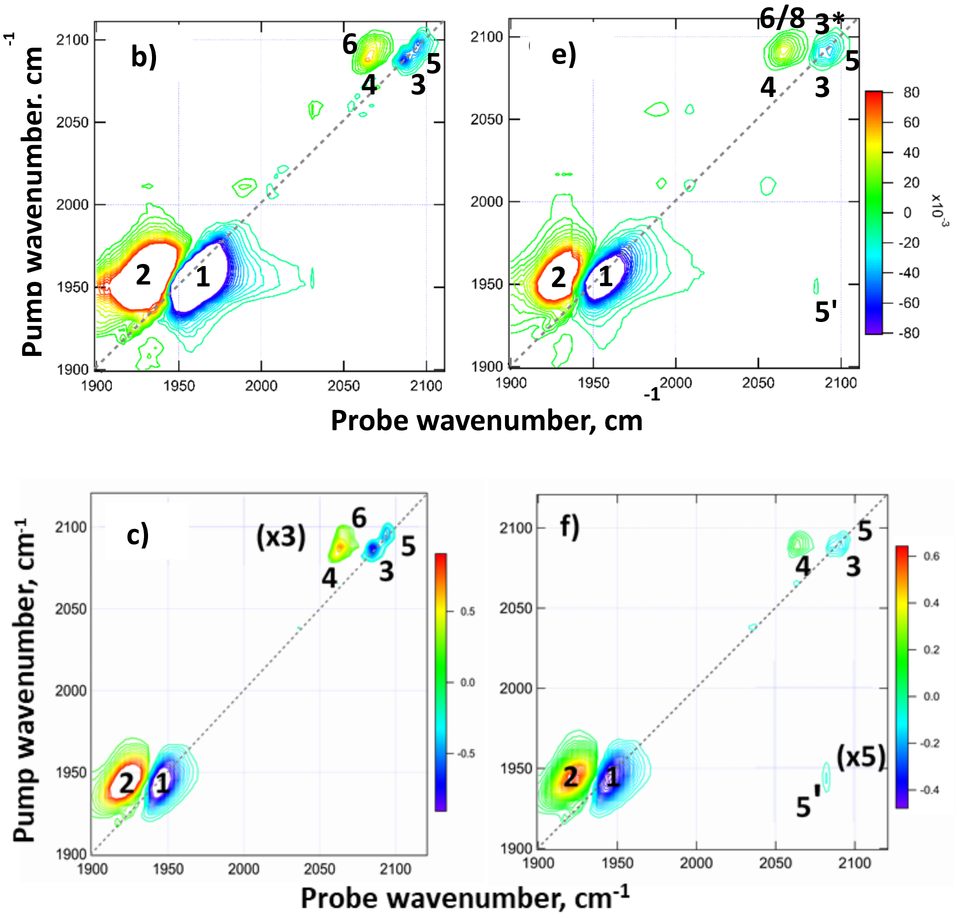

Figure S8. *2DIR spectra of **M1** at a T_w of 800 fs using parallel pump-probe polarization in a) D_2O; the grey box indicates an unknown impurity; b) CH_3CN; c) DMSO and perpendicular polarization d) D_2O; e) CH_3CN; f) DMSO.*

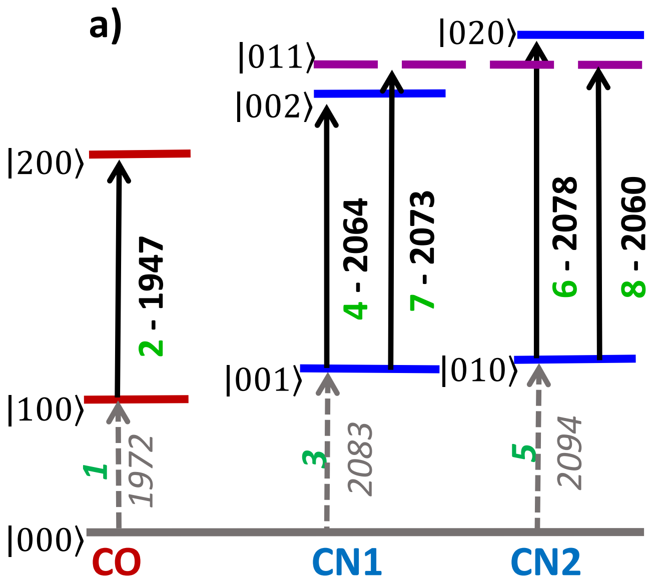

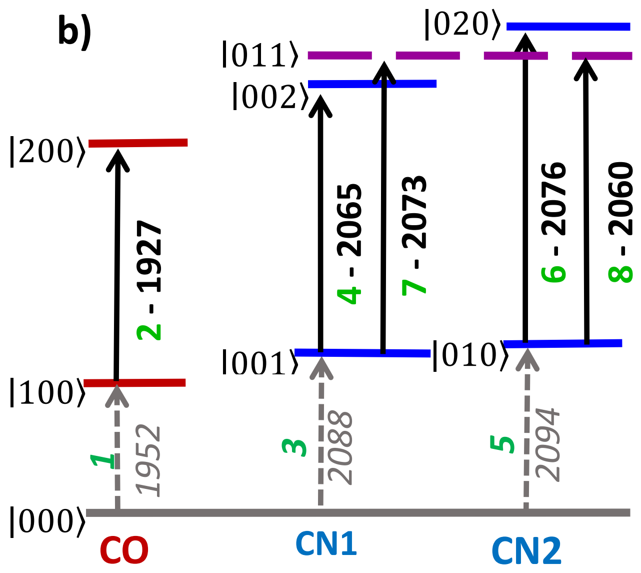

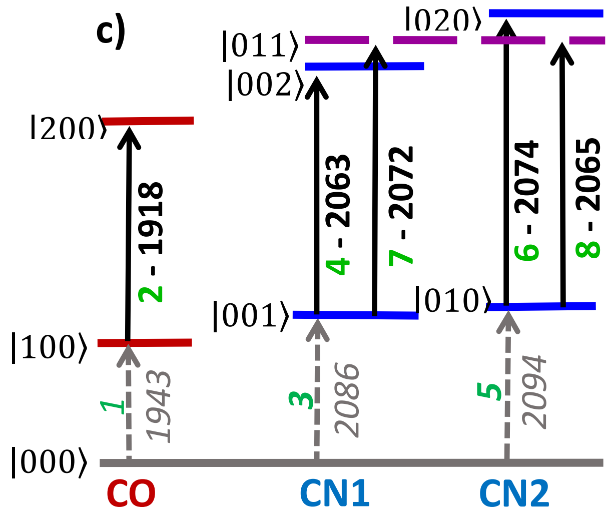

Figure S9. *Energy level diagram showing vibrational energy levels and transition energies of the ν_{CO} and ν_{CN} vibrational manifold, as detected for* ***M1*** *in a) MeOH, b) CH_3CN, c) DMSO. Transitions are labelled with numbers used to identify peak assignments in the 2D-IR spectra and text. Corresponding frequencies are reported alongside the arrows in cm^{-1}.*

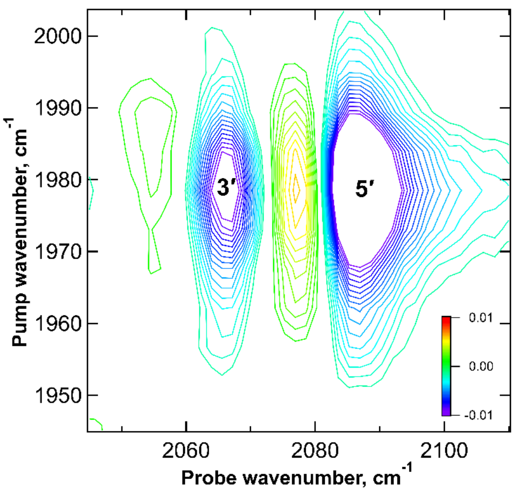

Figure S10. *Expansion of the ν_{CO}-ν_{CN} cross peak region of a 2D-IR spectrum for **M1** in H_2O at a T_w of 800 fs showing cross peaks 5' and 3' with their associated combination bands reflecting coupling strenght between the two modes. The spectrum was acquired at perpendicular pump-probe polarization.*

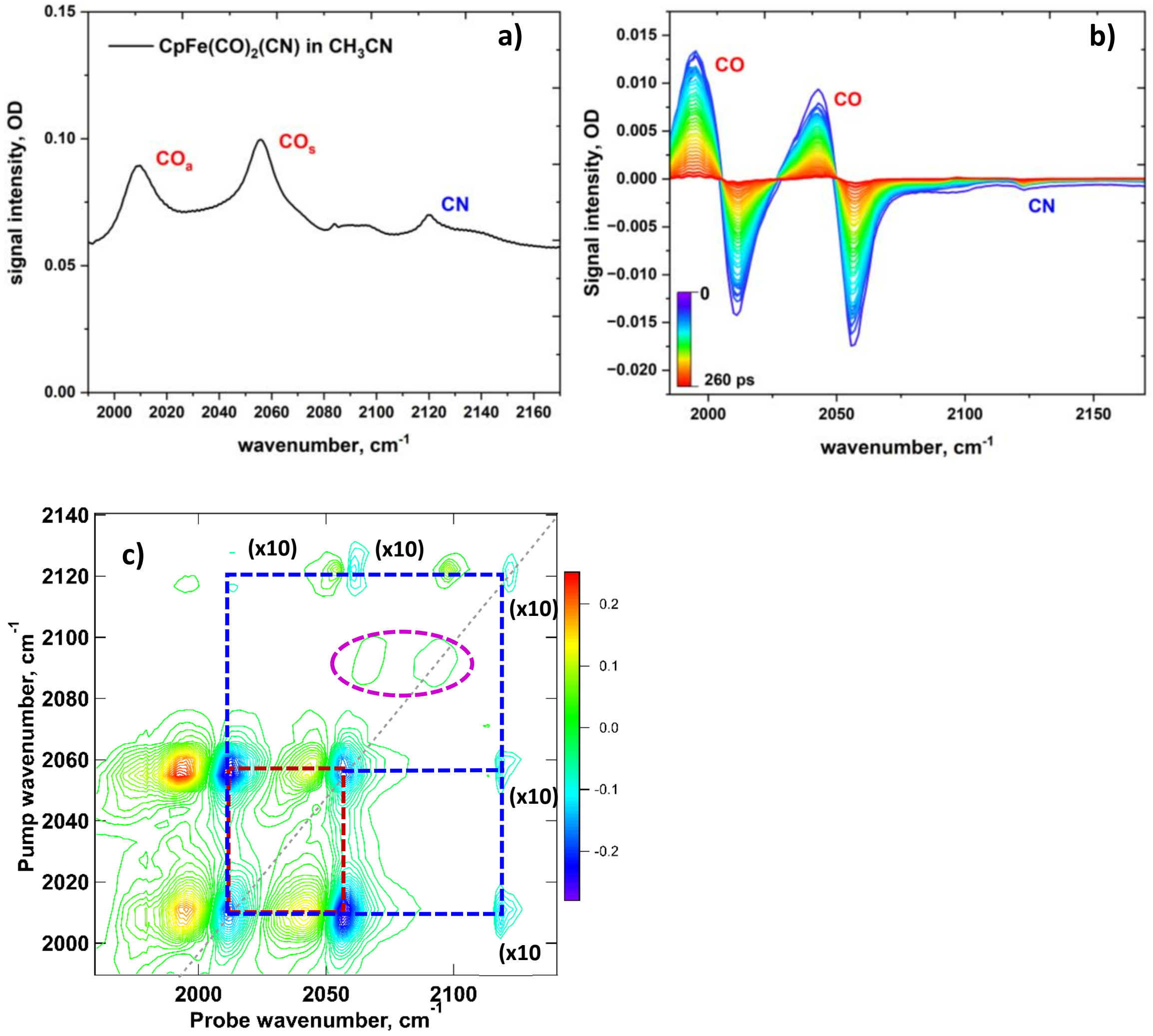

Figure S11. *a) FT-IR b) IR Pump- IR probe c) 2D-IR spectra of $CpFe(CO)_2(CN)$ in CH_3CN. The blue dotted rectangles highlight couplings between the ν_{CO} modes and the ν_{CN} modes, while the red one shows the*

*coupling between the asymmetric and symmetric ν_{CO} modes. The compound showed the presence of **M1** as impurity which is highlighted in the 2D-IR spectrum by the circle.*

Table S1. *IR frequencies (cm^{-1}) for the ν_{CO} and ν_{CN} modes of CpFe(CO)$_2$(CN) in CH_3CN, anharmonicities (cm^{-1}) and vibrational lifetimes (ps). CN1 refers to the antisymmetric stretch and CN2 refers to the symmetric one.*

	v = 0-1	v = 1-2	Intramode Anharmonicity	Intermode Anharmonicity	T_1, ps
CO_1	2010	1995	15	15	52 ± 3
CO_2	2056	2042	14	18	49 ± 3
CN	2120	2096	24	5	25 ± 5

Table S2. *IR frequencies for for the ν_{CO} and ν_{CN} modes of **M1** in mixtures of DMSO/H_2O obtained from Gaussian fitting of the experimental FTIR spectra. All spectroscopic values are given in cm^{-1}. CN1 refers to the antisymmetric stretch and CN2 refers to the symmetric one.*

DMSO/H_2O molecule ratio	ν(CO), *(FWHM)*	ν(CN_1), *(FWHM)*	ν(CN_2), *(FWHM)*	ν(CO) – ν(CN_1)	ν(CN_2) – ν(CN_1)
M1 *in DMSO*	1943 (22)	2086 (8)	2094 (7)	143	8
**1 : 0.2**	1946.3 (23)	2085.6 (11)	2095.4 (11)	139.3	9.9
**1 : 0.4**	1948.4 (24.)	2084.87 (12)	2095.5 (12)	136.5	10.6
**1 : 0.5**	1948.4 (25)	2084.7 (13)	2095.9 (12.5)	136.3	11.2
**1 : 0.6**	1947.1 (24)	2085.5 (12)	2095.8 (11)	138.5	10.3
**1 : 0.79**	1950 (25)	2084 (13)	2096 (13)	134	12
**1 : 2.36**	1958 (28)	2079 (16)	2093 (14)	121	14
**1 : 3.94**	1962 (29)	2077 (16)	2091 (15)	115	14
M1 *in H_2O*	1978 (20)	2067 (14)	2084 (12)	89	17

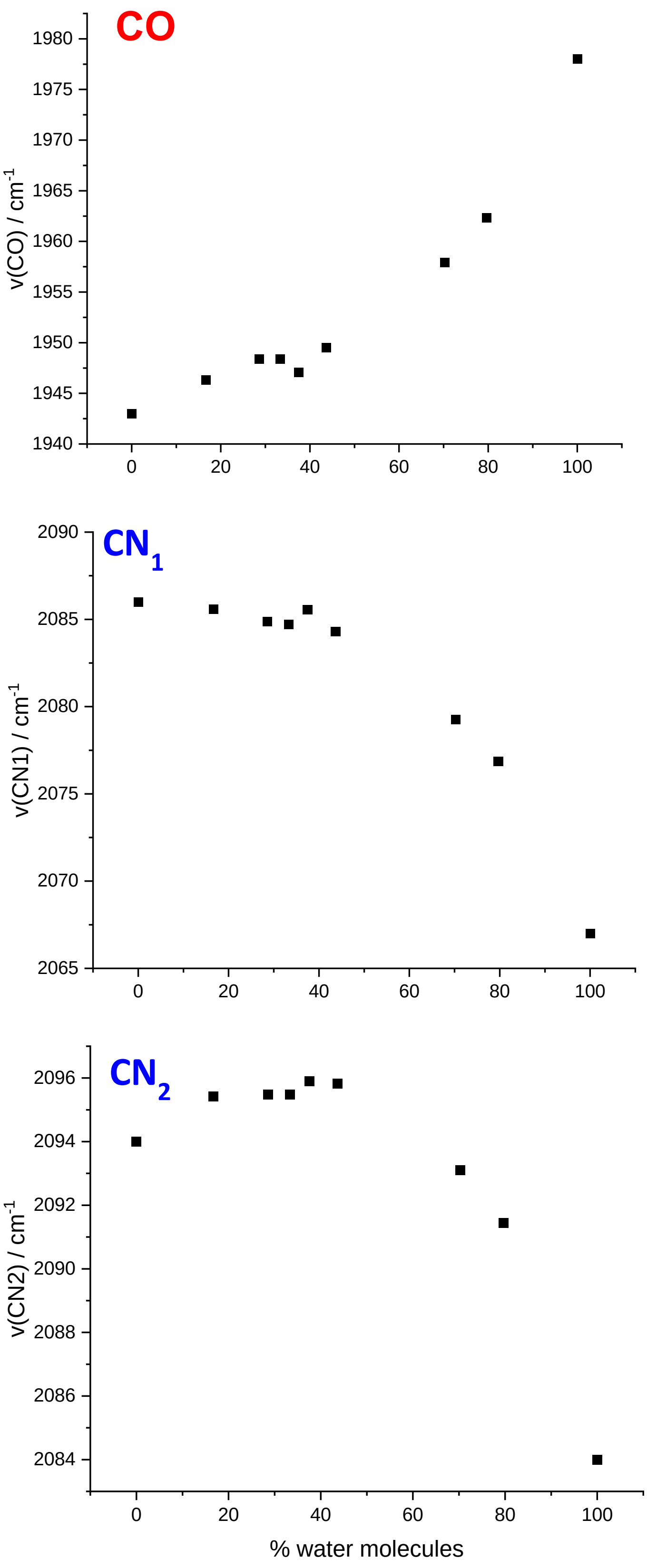

Figure S12. *Frequency shifts for the ν_{CO}, ν_{CN1} and ν_{CN2} modes of **M1** with increasing % of water molecules.*

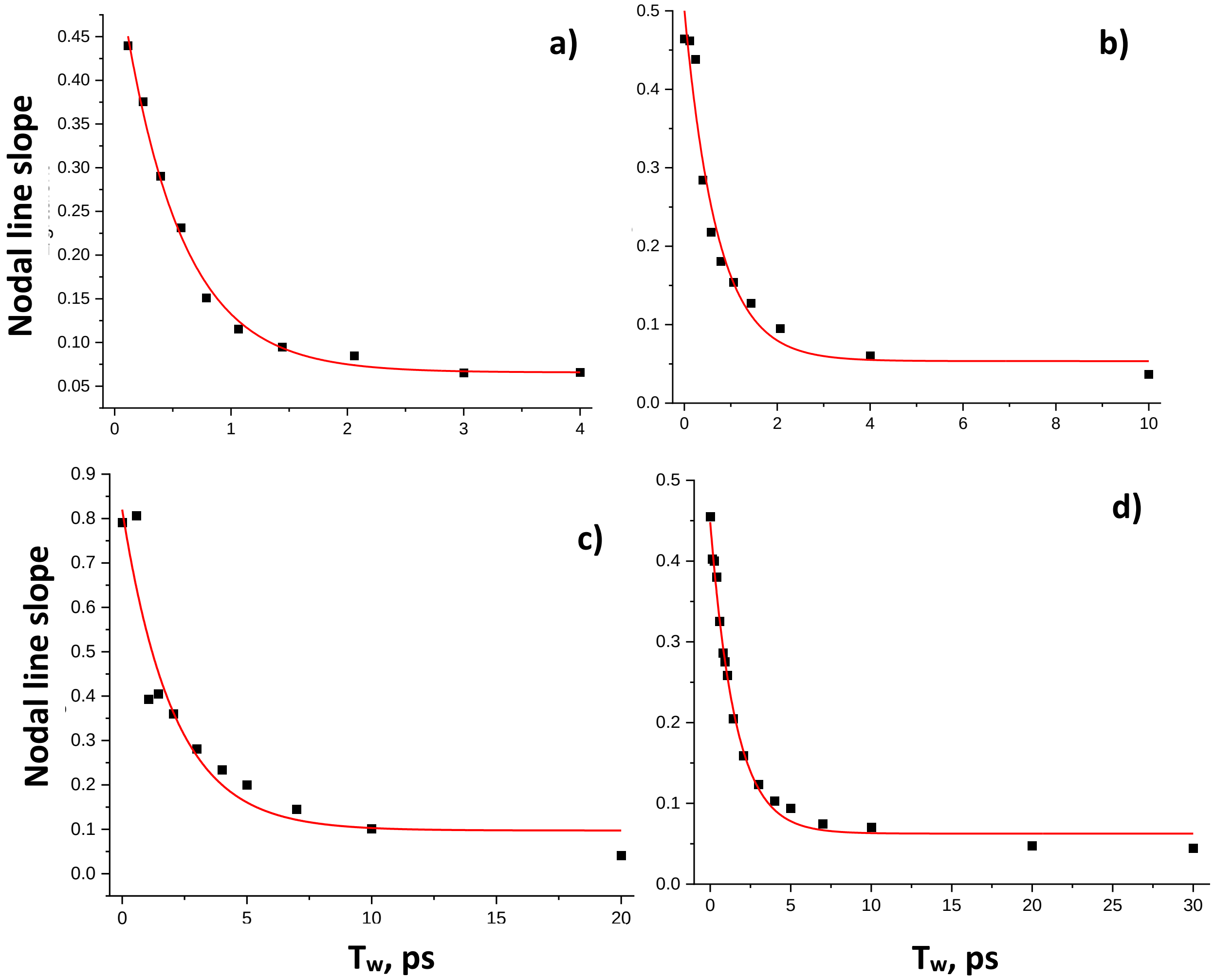

Figure S13. *Temporal dependence of the nodal line parameter to obtain the time-dependence measure of the frequency fluctuation correlation function for ν_{CO} mode of* ***M1****. The red line is a monoexponential fit to the experimental data. a)H_2O; b) D_2O, c) MeOH, d)DMSO.*

Table S3. *Spectral diffusion constants (ps) obtained in the different solvent by the nodal line slope method by fitting a monoexponential funtion to the data as shown in Figure S13.*

**Solvent**	**Spectral diffusion / ps**
H_2O	0.5 ± 0.1
D_2O	0.71 ± 0.12
MeOH	2.1 ± 0.5
DMSO	1.6 ± 0.1

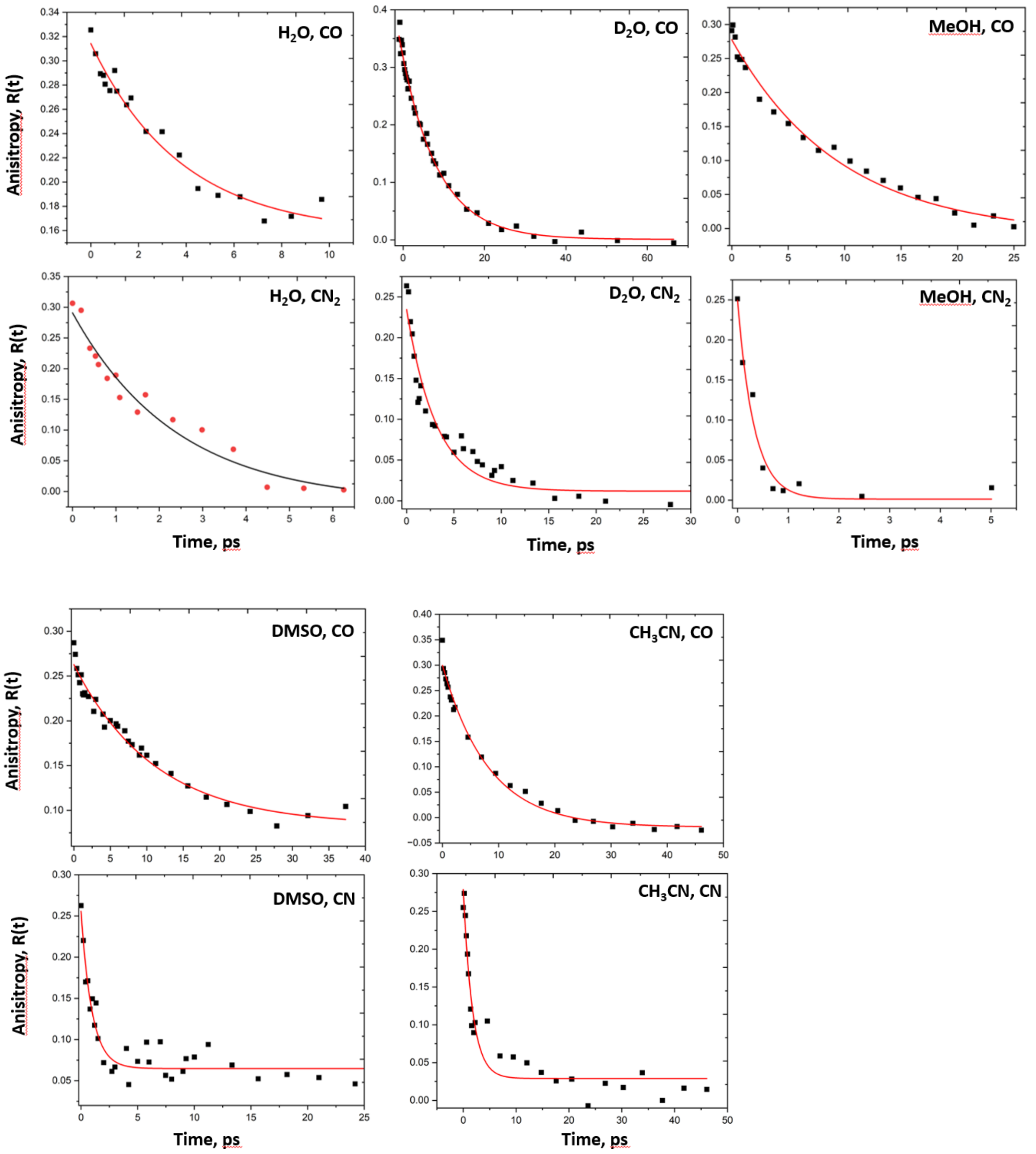

Figure S14. *Anisotropy decays for the CO and CN modes in the different solvents.*
